# A New Calcium(II)-Based Substitute for Enrofloxacin with Improved Medicinal Potential

**DOI:** 10.3390/pharmaceutics14020249

**Published:** 2022-01-21

**Authors:** Hou-Tian Yan, Rui-Xue Liu, Qi-Zhen Yang, Yan-Cheng Liu, Hong-Chang Li, Rui-Feng Guo, Lin-Hua Wu, Li-Min Liu, Hong Liang

**Affiliations:** 1 School of Chemistry & Pharmaceutical Sciences, State Key Laboratory for the Chemistry and Molecular Engineering of Medicinal Resources, Guangxi Normal University, Guilin 541004, China; yanhoutian@aliyun.com (H.-T.Y.); 2018110012@stu.gxnu.edu.cn (R.-X.L.); 2017010327@stu.gxnu.edu.cn (Q.-Z.Y.); 2019010318@stu.gxnu.edu.cn (H.-C.L.); 2018011084@stu.gxnu.edu.cn (R.-F.G.); 2 College of Pharmacy, Guangxi Medical University, Nanning 530021, China; wlh626@sohu.com (L.-H.W.); liulimin_gxykdx@hotmail.com (L.-M.L.)

**Keywords:** enrofloxacin, calcium(II) complex, veterinary drug, antibacterial activity, animal chemotherapy

## Abstract

Enrofloxacin (EFX) reacting with Ca(II) afforded a new complex, [Ca(EFX)_2_(H_2_O)_4_] (EFX-Ca), which was structurally characterized both in solid and solution chemistry. *E. coli* and *S. typhi* were tested to be the most sensitive strains for EFX-Ca. The LD_50_ value of EFX-Ca in mice was 7736 mg/kg, implying the coordination of EFX to Ca(II) effectively reduced its acute toxicity. EFX-Ca also decreased the plasma-binding rate and enhanced the drug distribution in rats along with longer elimination half-life. EFX-Ca also showed similar low in vivo acute toxicity and higher anti-inflammation induced by H_2_O_2_ or CuSO_4_ in zebrafish, with reactive oxygen species (ROS)-related elimination. The therapeutic effects of EFX-Ca on two types (AA and 817) of *E. coli*-infected broilers were also better than those of EFX, with cure rates of 78% and 88%, respectively. EFX-Ca showed promise as a bio-safe metal-based veterinary drug with good efficacy and lower toxicity.

## 1. Introduction

Common veterinary drugs play an important role in animal husbandry because of their preventive and therapeutic benefits. Antibacterial and bactericidal drugs against animal diseases account for a significant proportion of these veterinary drugs. Quinolones are a class of synthetic antibacterial drugs that have a characteristic structural skeleton of a 4-quinolone ring [1,2]. Since the nalidixic acid antibacterial agent was first discovered in 1962 in the United States by Lesher et al., quinolone-type antibacterial drugs have been extensively developed, studied, and used in the treatment of animal diseases [3,4,5]. The third-generation quinolone antibacterial drugs, named fluoquinolones (FQNs), were developed successfully in the 1980s. These FQNs not only introduce the fluorine group into the structural skeleton of quinolones (as 6-F) but also introduce the piperazinyl group [1,6]. Such structural improvements have enhanced drug penetration into the bacterial cells to increase the antibacterial efficacy of these drugs. This has significantly improved the bioavailability of such drugs in vivo and has extended the antibacterial range of FQNs compared with the previous two generations. Unlike other common antibiotics, FQNs target bacterial DNA and DNA gyrase [7,8]. They can effectively inhibit DNA cyclotron enzymes in bacterial cells, thus causing irreversible damage to bacterial DNA and blocking the division of bacterial cells [7,9]. In addition, FQNs are not affected by plasmid-mediated drug resistance and therefore have no cross-resistance to many antimicrobial drugs. Therefore, the cross-resistance between FQNs and other types of antimicrobial agents was not common for most plasmid-mediated bacterial resistance before the past two decades [10]. Some FQNs, such as enrofloxacin (EFX), lomefloxacin, and sarafloxacin, have been used clinically as veterinary drugs to treat animal diseases [11]. EFX is a typical representative of FQNs that is used specifically as a veterinary drug and shows a broad-spectrum antibacterial activity [12,13,14]. In addition, EFX shows an even better bactericidal effect and thus greatly reduces the toxicity to the central nervous system [15]. EFX can also create a suicidal factor or can destabilize GyrA dimer in bacteria, which induces chromosome fragmentation [16]. Conversely, EFX also has shown moderate acute toxicity and its most common side effects include inducing tendinopathy and cartilage damage and affecting the reproductive system. Furthermore, with a significantly increased application of this kind of antibiotic worldwide, especially in China, the inexorable increase in resistance, even cross-resistance, of these drugs has become a significant problem [17,18]. This is mainly due to the mutations in the gyrA gene, a target of FQNs, further followed by the enhancement of the active pumping mechanism of the bacterial cells [19]. Therefore, the improvement and regulation of these classic drugs are important aspects of drug improvement.

Globally, all types of metal complexes have been studied as potential antibacterial drugs for dozens of years and have shown their unique effects and prospects for treating diseases [20,21,22,23,24]. Some metal complexes of FQNs have also been synthesized and evaluated [25,26,27]. Unlike the original FQNs, some of these metal complexes have exhibited better therapeutic effects and higher inhibiting abilities of bacterial resistance. Nevertheless, it is still necessary to consider the toxic side effects with regard to the heavy metal centers of these metal complexes.

Calcium is one of the most important essential elements in the world, and it is also one of the indispensable elements in organisms. It plays an important role in the activities of life processes such as physiological activities, reproduction, immunity, and neural activity [28,29,30]. Compared with other transition metal complexes, far fewer reports have focused on the calcium complexes in the field of traditional metal complex research. This may be attributed to the fact that calcium has a saturated electronic structure that belongs to stearic acid, and as such, it tends to form ionic compounds, or calcium salts, but not calcium complexes [31]. Calcium plays an important role in the body. However, as proven in previous studies, the functioning of calcium in the body is closely related to the coordination binding state with different bio-ligands [32]. Therefore, calcium as a biosafe metal center especially in vivo has great appeal in the research and development of metal complexes and potential metal-based drugs.

Our previous work reported for the first time the calcium complex of a FQN drug (marbofloxacin). The results showed that this complex effectively reduced the toxicity in vivo while maintaining its antibacterial activity. The biological safety of the calcium complex of marbofloxacin suggested its medicinal potential. As a targeted potential veterinary drug, however, the achieved studies on this calcium(II) complex are not sufficiently in-depth. In addition, related pharmacokinetic studies and clinical therapeutic effects have also not been involved [32]. Conversely, the high price of marbofloxacin has resulted in the high expected cost of this calcium complex in clinical use, contributing to its poor market prospects as a potential veterinary drug. On the premise of maintaining similar antibacterial efficacy, EFX is only 1/20th of the cost of marbofloxacin, and its application in the veterinary market is predominant. Therefore, it is more attractive to study the synthesis and properties of a new calcium(II) complex of EFX, and to conduct pharmacokinetic studies and evaluate its clinical therapeutic effect for livestock on this basis, which has shown much better research value and application prospects. Therefore, in this study, we synthesized and structurally characterized EFX, a new calcium(II) complex of EFX, as a more promising metal-based substitute for the classic FQNs in potential clinical applications. To explore its medicinal prospects as a new type of metal-based veterinary drug, we conducted preferential experiments on the bacteriostatic, acute toxicity, and pharmacokinetic effects, as well as its quasi-clinical therapeutic effects, on broilers in comparison with EFX.

## 2. Results and Discussion

### 2.1. Structural Characterization of EFX-Ca

**Figure 1:**
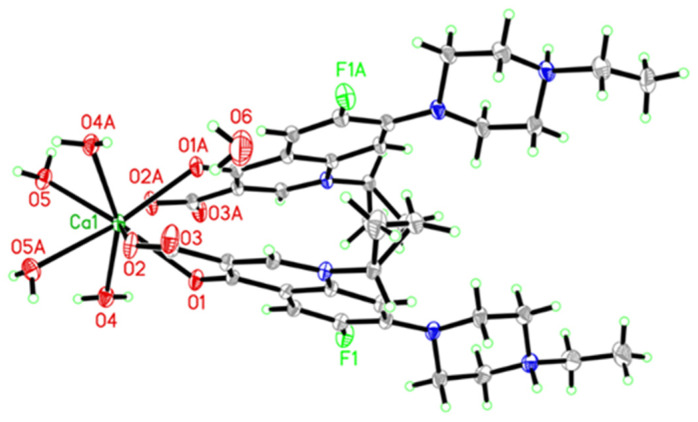
The ORTEP drawing for the crystal structure of EFX-Ca.

As shown in Figure 1, the calcium(II) complex of EFX, [Ca(EFX)_2_(H_2_O)_4_]·H_2_O (EFX-Ca), crystalized in a monoclinic system with space group *C2/c*. The central Ca(II) adopted an eight-coordinated geometry, in which two deprotonated EFX chelated to Ca(II) by the 4-carbonyl O and the deprotonated 3-carboxyl O. Two EFX located in the *cis*- form, and four H_2_O molecules coordinated to Ca(II) on the other side to form a dodecahedral configuration. Furthermore, the whole complex crystallized in the form of mirror symmetry. Thus, one EFX ligand and two water molecules located at the position of the mirror images of another EFX ligand and the other two water molecules of this dodecahedral complex, with the same bond lengths and bond angles. As shown in Supplementary Materials Figure S1 (ESI†), the packing diagram of the crystal structure of EFX-Ca viewed along the b-axis of the unit cell demonstrated the three-dimensional supramolecular structure of EFX-Ca consolidated by the C–H···O hydrogen bonding and π–π stacking interactions between the neighboring EFX ligands. Note also that the entire EFX-Ca molecule formed as electrically neutral based on the deprotonation on the 3-carboxyl group of each EFX ligand. The primary crystallized data and the refinement parameters for the crystal structure of EFX-Ca are given in Table 1. The selected bond lengths (Å) and bond angles (°) for EFX-Ca are listed in Table S1.

### 2.2. Determination on the Water Solubility

**Figure 2:**
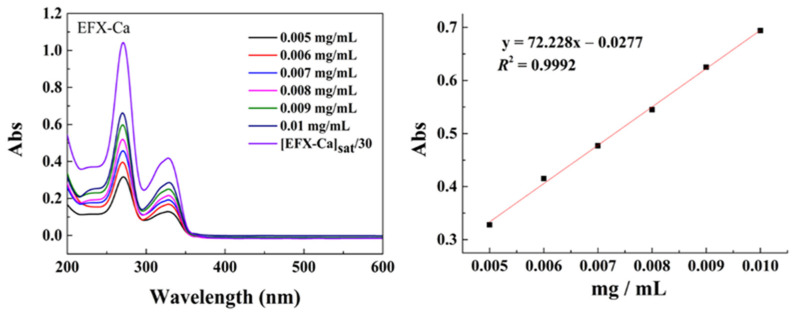
The water solubility of EFX-Ca at room temperature was determined by the Lambert–Beer law based on the standard line of the water solubility of EFX-Ca derived from a series of determined concentrations. The concentration of the working solution of EFX-Ca indicated by the UV-Vis spectrum was based on a 30× dilution on the saturated aqueous solution of EFX-Ca.

Furthermore, we also examined the water solubility of EFX-Ca, in comparison with EFX, by UV-Vis spectral analysis of the prepared aqueous solution, based on the Lambert–Beer law. As shown in Figure S2 (left) (ESI†), we found that a 30× dilution of the saturated aqueous solution of EFX gave the peak absorbance at 273 nm. According to the Lambert–Beer law based on the standard line of EFX derived from a series of working solutions prepared in different concentrations of EFX (Figure S2 (right) (ESI†)), we determined the water solubility of EFX to be 0.2042 mg/mL. Using the same measuring method based on the Lambert–Beer law, the coordinated EFX-Ca showed a more satisfying water solubility, as shown in Figure 2. Thus, we calculated and determined the value of the water solubility of EFX-Ca to be 0.46 mg/mL, which was more than twice that of EFX. The enhancement of the water solubility of EFX-Ca could be attributed partly to the formation of the charged species of EFX-Ca, as suggested by the results of ESI-MS. In practical applications, moderate water solubility is important for veterinary drugs, so this result provided a positive result for EFX-Ca and holds significant benefits for its potential druggability index.

### 2.3. In Vitro Antibacterial Activity Study

The MIC and MBC values of EFX-Ca were further evaluated from in vitro antibacterial activity screening. Results of antibacterial data are listed in Table 2, which show that EFX and EFX-Ca exhibited similar antibacterial activities toward all five tested bacteria. The tested bacteria are common pathogens in humans, including *Staphylococcus aureus*, *Escherichia coli*, *Salmonella typhi*, *Pseudomonas aeruginosa*, and *Proteus vulgaris*.

Table 2 shows that EFX and its metal complexes for five pathogenic bacteria all had different degrees of bacteriostatic and bactericidal effects on bacteria. Among them, they had the best antibacterial effect against *E. coli* and *S. typhi*, followed by bactericidal action against *P. aeruginosa* and *P. vulgaris*, and the worst resistance to *S. aureus*. In addition, by comparing the ligands and complexes, it was evident that EFX and its metal complexes were quite similar to the MIC and MBC of five pathogenic bacteria with a slight change. This tiny change, however, indicated that the antibacterial effect of the EFX-Ca on *E. coli* was stronger than the ligand. EFX-Ca effectively retained the typical antibacterial activity of EFX after coordination with Ca(II), and appropriately improved the antibacterial ability against certain bacteria.

### 2.4. In Vivo Acute Toxicity in Mice

#### 2.4.1. Drug Effect on the Body Weight of Mice

**Figure 3:**
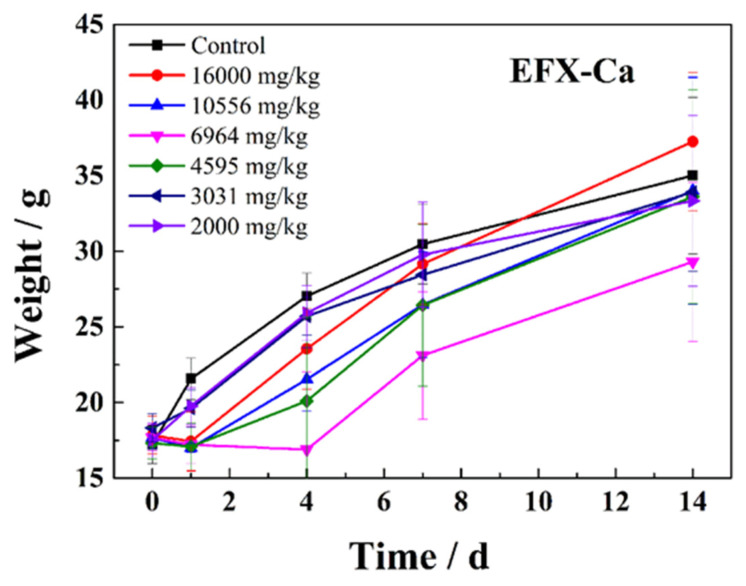
The time-dependent effects on the body weight of the tested KM mice after the oral administration of EFX-Ca at different dosages (2000, 3031, 4595, 6964, 10,556 and 16,000 mg/kg).

Based on the satisfying in vitro antibacterial activity of EFX-Ca indicated by the above MIC and MBC values, the in vivo tests for the biological activity of EFX-Ca were further carried out. In general, the acute toxicity test is the primary step in testing drug toxicity, and mice are the most commonly used target animals to assess this factor. So the acute toxicity of EFX-Ca, compared with EFX and EFX-Na, was firstly tested. The results of the influence on the body weight of mice under the treatment of EFX-Ca, EFX and EFX-Na, are listed in Table 3, Tables S2 and S3 (ESI†) as well as in Figure 3, Figures S3 and S4 (ESI†). According to the results, except for the 16,000 mg/kg, which could reduce the body weight of the mice on the first day (D1), the doses of EFX did not cause significant changes in body weight after administration, compared with the control group. In addition, at the end of the experimental observation period, the body weight of the mice was not significantly different from that of the control group. These results indicated that only high doses of EFX in aqueous solution affected the body weight of KM mice, and these effects were reversible over time. The results for the effect of EFX-Na on the body weight were comparable to those of EFX, but under much lower doses in the range of only 500–2000 mg/kg. Relative to the EFX results, EFX-Ca deeply affected the body weight of the mouse. Table 3 shows that the different doses of EFX-Ca caused the body weight of KM mice to decrease at the first day (D1) in groups 1–4 after administration. In addition, this trend of changes on the body weight had a certain relationship with the dosage of each dose group. The body weight for each group was enhanced again in a time-dependent mode. The effect of the EFX-Ca on the body weight of the tested mice was basically equal to that of EFX or EFX-Na. Thus, we concluded that the toxicity of EFX-Ca relative to its influence on body weight was as satisfying as that of EFX.

#### 2.4.2. Effect of Mice Mortality

The relationship between the number of deaths in mice and the concentration of medication under the acute toxicity experiments is given in Table 4. Although EFX has been used as a common antibacterial drug for some time and has been tested for effectiveness and safety, we repeated the mortality test to determine a comparative role. We found that the number of surviving KM mice substantially increased when the dose of the drug was reduced, and the number of surviving KM mice in each dose group gradually reduced over time. Under the same dose, EFX-Ca showed significantly lower acute toxicity than EFX. This result clearly showed that under a dosage ranging from 2000 to 10,556 mg/kg, the number of mouse deaths caused by the ligand was one or two more than the number of mouse deaths caused by the complex (out of 10). Note that the EFX-Ca did not cause death in mice at a concentration of 2000 mg/kg, whereas the ligand caused the death of two mice at the same dosage.

According to the improved Karber’s method, we calculated the LD_50_ value for EFX-Ca, EFX, and EFX-Na to be 7736 mg/kg, 5312 mg/kg, and 1421 mg/kg with a 95% confidence interval of 5274.73–11,371.04 mg/kg, 3543.24–7954.26 mg/kg, and 1136.84–1755.09 mg/kg, respectively. This further proved that the coordination of calcium ions effectively reduced the toxicity of EFX. Thus, through the lethality, LD_50_, and the linear relationship between body weight and time after administration, we further verified that the tested complexes showed a significant reduction in acute toxicity in vivo compared with EFX alone.

### 2.5. Histopathological Examination

**Figure 4:**
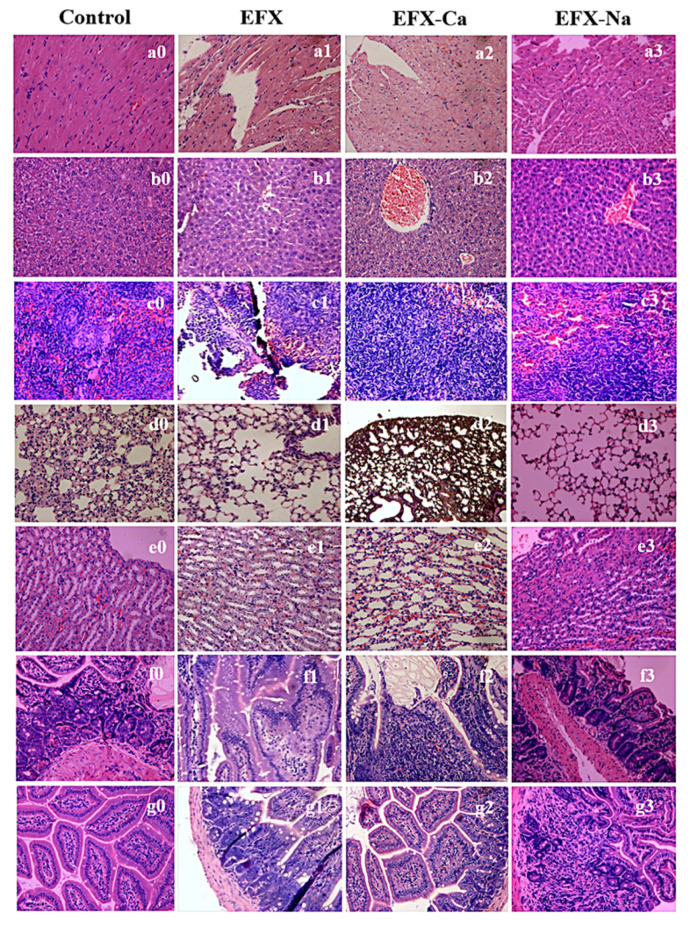
Histopathological images under 40× magnification on the main organs from the dead mice after the oral administration of EFX, EFX-Na, and EFX-Ca, in which the histologic tissue slices of (**a**) heart, (**b**) liver, (**c**) spleen, (**d**) lung, (**e**) kidney, (**f**) large intestine, and (**g**) small intestine were stained and visualized. Number 0, 1, 2, 3 indicates the effect of EFX, EFX-Ca and EFX-Na for each organ, respectively.

On histopathological examination of the mice, there was no intra-abdominal fluid and the organs were in a normal position. Most organs, including the heart, liver, spleen, lung, kidney, large intestine, and small intestine, did not show changes in color, texture, or volume, which indicated a lack of obvious abnormalities and lesions. Subsequently, we compared histopathological observations of the tissue slices, as shown in Figure 4. Viewed from Figure 4a–g, respectively (for the heart, liver, spleen, lung, kidney, large intestine, and small intestine), in each high-dose group, the tissues and structures of the heart, liver, spleen, large intestine, and small intestine of the dead mice were basically normal, and the cells were stained uniformly, which indicated that the high-dose drug had no obvious toxic and side effects on these organs. In the high-dose group, EFX-Ca resulted in significant inflammatory cell infiltration of the lung tissue. EFX-Na caused more and larger glomeruli in kidney tissue, and EFX-Ca led to the enlargement of the renal capsule, which was accompanied by renal edema and lipid formation. This result suggested that the main target organ for the toxic side effects of EFX-Na was the kidney, whereas EFX-Ca had a slight effect on the kidney and lung.

### 2.6. Pharmacokinetic Study on EFX-Ca

It is necessary to study pharmacokinetics and pharmacodynamics (PK-PD) as one of the indexes to evaluate the medicinal properties of compounds. Therefore, PK-PD determination of EFX-Ca based on animal blood tests and analysis was further performed in rats, since the noninvasive blood collection in rats is more convenient than that in mice.

#### 2.6.1. Establishment of Standard Curve and the Minimum Concentration for Administration

In the range of drug concentration from 0.05 to 1.0 μg/mL, the chromatographic peak area had a good linear relationship with the concentration of each standard working solution (R^2^ = 0.9997), and we obtained the linear curve of the standard curve as *y* = 19,373*x* − 72.512. In addition, the lowest concentration that we detected was 0.025 μg/mL. Table S4 and Figure S5 (ESI†) provide the details.

#### 2.6.2. Pharmacokinetic Analysis

**Figure 5:**
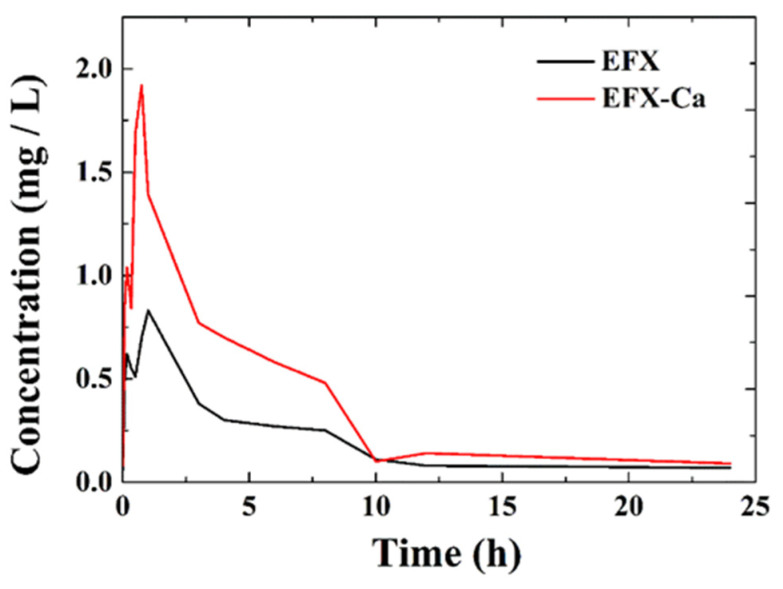
The relationship between drug concentration and the elimination time in plasma samples from the tested SD rats treated with EFX-Ca and EFX at different time points (0, 0.083, 0.333, 0.5, 0.75, 1, 3, 4, 6, 8, 10, 12, and 24 h).

The plasma samples collected at different time points for the pharmacokinetic properties of EFX-Ca compared with EFX were examined and analyzed accordingly. According to the specification curve, we could record the peak areas and all of the plasma concentrations at different time points, through which the average drug–time curve could be drawn (as shown in Figure 5). The drug concentrations in the plasma of SD rats for EFX-Ca and EFX at different time points after administration are given in Table S5 (ESI†). By means of data manipulation and analysis in silico, we obtained a series of primary pharmacokinetic parameters (as listed in Table 5).

As shown in Table 5, we calculated the apparent volume of distribution Vd to be 1053.16 mL/kg, after we orally administered EFX (10 mg/kg) to SD rats. The highest plasma concentration was 830 ng/mL, the peak time was 1 h, the half-life of elimination was 2.43 h, and the area under the drug-time curve (AUC) was 2702.50 ng·h/mL, with a clearance rate of 37 mL/kg·h. These parameters indicated that EFX could be rapidly and widely distributed and eliminated slowly in SD rats. Comparatively, under the same experimental conditions, after we orally administered EFX-Ca (12 mg/kg) to SD rats, the apparent volume of distribution Vd was 267.44 mL/kg, the highest plasma concentration was 1920 ng/mL, the peak time was 1 h, the half-life of elimination was 4.41 h, and the AUC was 5875.83 ng·h/mL, with a clearance rate of 20.42 mL/kg·h. We also found that EFX-Ca could be rapidly and widely distributed and slowly eliminated in SD rats.

After oral administration of the same effective dose of the drug in SD rats, the parameters not only indicated that the long half-life and the widely rapidly distributed metabolic characteristics were the same, but also showed the difference between EFX and EFX-Ca by comparing the main pharmacokinetic parameters. To a certain extent, the pharmacokinetic characteristics of EFX were different from EFX-Ca. The AUC of EFX-Ca was 5875.83 ng·h/mL, which was more than twice that of EFX (2702.50 ng·h/mL). The results also demonstrated that the coordination of EFX with Ca(II) could increase the total amount of drug into the systemic circulation. In addition, the highest plasma concentration of EFX was 830 ng/mL, which was lower than that of EFX-Ca (1920 ng/mL), but its peak time (1 h) was higher than EFX-Ca (0.75 h). This result indicated that the binding rate of EFX to plasma protein in rats was higher than the EFX-Ca, but it naturally reduced its bioavailability in animals. The production of its calcium complex effectively alleviated this situation. Furthermore, the half-life of elimination of EFX was tested to be 2.43 h, which was lower than that of EFX-Ca. Therefore, this result indicated that EFX could prolong the drug effect after coordinating with the bioactive calcium(II), and finally, it could improve drug utilization.

### 2.7. In Vivo Acute Toxicity and Anti-Inflammatory Effects on Zebrafish

As a small tropical freshwater fish, zebrafish has the characteristics and advantages of high homology with mammals, short development and growth cycle, autotrophic development within one week, etc. [33]. As a result, zebrafish have been developed into a mature and efficient drug screening platform [34,35]. In order to more comprehensively study the potential toxicity and anti-inflammatory effects of EFX-Ca, the zebrafish system and its two typical inflammatory models respectively induced by CuSO_4_ and H_2_O_2_ were also used to compare the toxicological properties between EFX-Ca and EFX.

#### 2.7.1. The In Vivo Acute Toxicity in Zebrafish

The acute toxicity of EFX-Ca, compared with EFX and EFX-Na [36], was primarily tested in the zebrafish incubation system, from which the developmental status or the potential physiological abnormalities of the zebrafish, treated by medicated bath of different dosage of EFX-Ca and EFX, respectively, were monitored and examined to provide greater understanding and insight into the potential alteration on the toxicity of the Ca(II)-based enrofloxacin in vivo.

A concentration range from 0.01 to 20 μM and an incubation time range from 24 to 120 hpf were adopted to give a more legible assessment for the tested compounds [36]. The experimental results are shown in Figures S6 and S7 (ESI†), in which the zebrafish showed no obvious physiological abnormalities under the incubation with the full-range concentration of EFX-Ca. However, the embryonic development rate of zebrafish decreased gradually in a dose-dependent manner, while the body length of zebrafish was also slightly affected after the medicated bath compared to our previously reported copper(II) complex of enrofloxacin, EFX-Cu [36]. The acute toxicity of EFX-Ca, as well as EFX, to the zebrafish embryos and larvae was significantly reduced at the same concentration, suggesting a much lower acute cytotoxicity of this Ca(II)-based enrofloxacin. On the other hand, similar to EFX-Na [36], when EFX-Ca or EFX were given at a higher concentration, the fetal membrane of the zebrafish embryos sometimes appeared villous or flocculent. The statistical data of survival and death of zebrafish after incubation with different concentrations of EFX-Ca or EFX are shown in Table 6.

#### 2.7.2. The In Vivo Anti-Inflammatory Effects Induced by CuSO_4_ in Zebrafish

With the transgenic Tg(mpx:eGFP) zebrafish as the research object, the process of inflammatory cell aggregation and migration in zebrafish can be easily observed in a non-invasive real-time dynamic manner, especially through this type of transparent juvenile zebrafish, so as to compare the anti-inflammatory effects of EFX-Ca and EFX.

**Figure 6:**
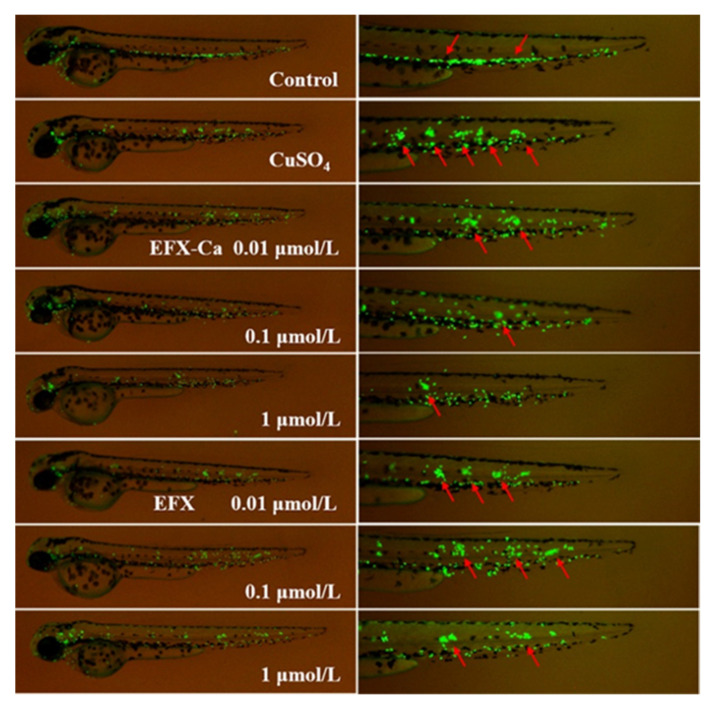
The inhibition effect of EFX-Ca, compared with EFX at the same concentrations (0.01, 0.1, 1 μM), on the observed neutrophil cluster aggregation under the CuSO_4_-induced mode of the transgenic zebrafish line Tg(mpx:eGFP). The partial magnified images of the latter part of the zebrafish were shown on the right side. The red arrows in the right side represent aggregations of neutrophils.

**Figure 7:**
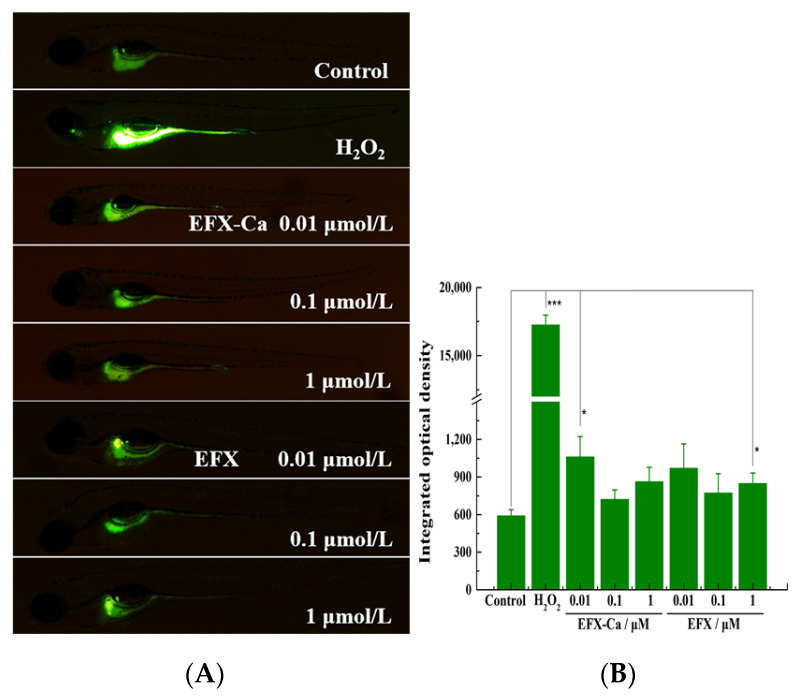
(**A**) The anti-inflammatory effect of EFX-Ca, compared with EFX at the same concentrations (0.01, 0.1, 1 μM), on the H_2_O_2_-induced ROS production in zebrafish model, indicated by the green fluorescence probe and visualized by the added DCFH-DA. (**B**) The quantitative results for the oxidative stress in zebrafish, represented by the histogram on the right side, were statistically analyzed based on examining the integrated optical density (IOD) of the green fluorescence in the tested zebrafish. Mean ± SD: * *p* < 0.05, *** *p* < 0.001.

Firstly, by using the GFP-labeling method to observe the zebrafish in the control group, it was found that neutrophils were evenly distributed in the body of zebrafish, and did not gather in clusters, as shown in Figure 6 [37,38,39]. On the contrary, in the tested zebrafish induced by CuSO_4_, neutrophils were obviously observed to migrate and eventually cluster together [37,38,39]. After pre-incubation with EFX-Ca or EFX at different concentrations for 1 h, the tested zebrafish showed varying degrees of resistance to CuSO_4_-induced neutrophil aggregation. Although the aggregation of neutrophils could not be completely inhibited at lower concentrations, there was a certain difference between EFX-Ca and EFX with the increasing concentrations of the medicated bath. For EFX, partial aggregations of the neutrophils could be observed in zebrafish even after the pretreatment at the maximum concentration of 1 μM. Under the same concentration of EFX-Ca, the neutrophil aggregation was more effectively inhibited. Especially in the presence of EFX-Ca at a lower concentration of 0.1 μM, neutrophils in zebrafish could be observed to be significantly dispersed, while neutrophils of the EFX-treated group under the same concentration still maintained obvious clustering status, indicating that EFX-Ca has a stronger anti-inflammatory effect on CuSO_4_-induced zebrafish than EFX, with a certain concentration-dependence. It should be noted that 10–15 zebrafish were set and treated in each group, and three of them were chosen randomly for examination for each group. Only one of the three zebrafish in each group is shown in Figure 6, so that the results could be demonstrated more characteristically. While the other two examined zebrafish with similar results in each group were compared and are together shown in Figures S8 and S9 (ESI†). The following anti-inflammatory results for the H_2_O_2_-induced zebrafish were also shown in this manner, respectively in Figure 7, Figures S10 and S11 (ESI†).

#### 2.7.3. The In Vivo Anti-Inflammation Effects Induced by H_2_O_2_ in Zebrafish

According to the above results based on the CuSO_4_-induced inflammation model in zebrafish, EFX-Ca and EFX both showed anti-inflammatory effects to varying degrees. In the early stage of inflammation, oxidative stress in the injured site was activated and a large amount of ROS was released. Therefore, in order to further confirm whether EFX-Ca could exhibit the antioxidant effect, H_2_O_2_ was also selected to induce oxidative stress in zebrafish, and the green fluorescent probe (DCFH-DA) was used for the visual detection [36]. The experimental results are shown in Figure 7, Figures S10 and S11 (ESI†).

By comparing the blank group with the H_2_O_2_-induced oxidative stress group, it can be seen that after the addition of DCFH-DA probe, the green fluorescence in the H_2_O_2_-induced group was significantly enhanced, compared with the blank group, indicating that the zebrafish had produced an obvious oxidative stress reaction in vivo. However, when comparing the zebrafish that received the same H_2_O_2_ stimulation with and without the 48 h of pre-treatment with the medicated bath (EFX-Ca or EFX), the green fluorescence was significantly weaker than that of the positive group induced by H_2_O_2_ (with the average IOD (Integrated Optical Density) = 17,250 a.u.). Especially in the 1 and 0.1 μM medicated bath groups, the green fluorescence was very weak, with corresponding average IODs of 863 and 721 for EFX-Ca, and 848 and 772 for EFX, respectively, which was basically very close to that in the un-induced control group (average IOD = 590), indicating that both EFX-Ca and EFX could effectively resist the production of ROS in zebrafish when stimulated by H_2_O_2_. Even at the lowest concentration (0.01 μM) of EFX-Ca or EFX, the green fluorescence was also greatly reduced to the average IOD of 1060 for EFX-Ca and 970 for EFX, respectively. All of the average IOD ± SD values for statistical analysis are summarized in Table S6. In conclusion, both EFX-Ca and EFX can effectively resist or remove the H_2_O_2_-induced ROS production, and they also show an overall concentration dependence. Comparatively, EFX-Ca has a slightly stronger anti-ROS production ability than EFX, which could be proved from the results of all three concentration groups. This result is consistent with the results of the CuSO_4_-induced inflammation model in zebrafish.

Overall, the above experimental results based on the zebrafish platform have clearly indicated that EFX-Ca showed satisfying anti-inflammatory activity in vivo, and the anti-inflammatory effect is slightly better than EFX, which will be an important basis for the development of EFX-Ca as a potential metal-based veterinary drug, and will also provide a mechanism for the following in vivo experiments of bacterial infection-treatment evaluation. On the other hand, EFX-Ca and the original drug, EFX, showed similar in vivo toxicity in zebrafish, which was different from the distinguishing acute toxicity between EFX-Ca and EFX evaluated in mice. However, both of the different in vivo acute toxicity assessments showed that the toxicity of EFX-Ca was not higher than that of enrofloxacin and had ideal drug safety.

### 2.8. Quasi-Clinical Trial on Two Types of Infected Broilers

EFX is one of the most commonly used therapeutic drugs in the veterinary drug market. It has the characteristics of broad spectrum antibacterial activity, significant efficacy and low price [12,13,14]. EFX-Ca, as a potential alternative to EFX, has also been proven to maintain broad spectrum antibacterial activity while keeping the same cost, especially for G-type bacteria, such as *E. coli*, so it is indeed necessary to evaluate whether the efficacy of EFX can be achieved or even higher in the animal clinic. Therefore, we further investigated the therapeutic effect of EFX-Ca on bacterial infection in chickens, which are commonly used for EFX treatment.

#### 2.8.1. General Clinical Observations on the Infected Chickens

The mental state, food intake, water intake, and excretion of feces were normal in the healthy control group that was not infected and not given the drug. In each administration group, a certain number of chickens died in the first two days after administration, accompanied by different degrees of depression and lethargy, a sharp decline in food intake, and increased water intake. Additionally, these chickens occasionally issued a hoarse “gurgling” sound and exhibited other clinical symptoms. Compared with the uninfected chickens that showed normal symptoms and excreted normal feces (Figure S12A,B), most of the infected chickens appeared isolated, motionless, or gathered and curled up in a corner, with eyes closed or half open and dozing, head buried under wings, wings drooping, unkempt feathers, and other symptoms (see Figure S12C). The chickens that were administered EFX-Ca also excreted loose feces, which were mostly green, yellow-green, or in white liquid, and sometimes had blood stool or egg-white-like feces, and the feathers near the anus were often contaminated with feces (Figure S12D,E). Some chickens were severely dehydrated because of the diarrhea caused by pathogenic bacteria and inadequate drinking (Figure S12F). On day 3 following the administration of each dose group, the mental state of some chickens improved significantly, and they could walk freely and began to eat and drink freely. The feces changed from thin to soft, from yellow-green egg-white to gray-black column or lump, and a small amount of white urate could also be found. The intake of chickens began to increase, and the number of deaths of chickens gradually decreased. On days 4–5 following the administration of the drug, a small number of infected chickens still died, and some of the chickens curled up, were depressed, and excreted yellow-green loose feces. By day 6 following administration, most of the chickens were able to return to a normal state.

We observed the pathologic anatomy of the tested chickens in the healthy control group (not infected, not given) and compared this with those that died in the infected group without drug administration. We found that the diseased chickens that died showed marked symptoms of perihepatic inflammation, pericarditis, balloon inflammation, omphalitis, ophthalmitis, articular synovitis, hemorrhagic enteritis, and granuloma, as well as dehydration resulting from diarrhea. Detailed autopsy of some tissues and organs are shown in Figure S13.

#### 2.8.2. The In Vivo Therapeutic Effect on the Infected Chickens after Drug-Mixed Feeding

We conducted quasi-clinical trials to examine the therapeutic effects of both EFX and EFX-Ca on the broilers infected by *E. coli*. EFX and EFX-Ca at high, medium, and low doses for two types of regular broilers (AA white feather broiler chickens (AA) and 817 mixed broiler chickens (817)) infected with *E. coli* were analyzed and compared. As shown in Table 7 and Table 8, the experimental results showed that under the same feeding conditions (temperature, light, and ventilation), a single dose of EFX and different doses of EFX-Ca had significant therapeutic effects on AA white feather broiler chickens and 817 broiler chickens (with stronger disease resistance) that were infected with *E. coli* and the therapeutic effects varied with different drugs and doses. For the AA white feather broiler, the therapeutic effect of different doses was as follows: EFX-Ca (medium dose) > EFX > EFX-Ca (high dose) > EFX-Ca (low dose), with the corresponding cure rates of 78%, 62%, 60%, and 54%, respectively. For the infected 817 broiler chickens, the therapeutic effects of each dose group were as follows: EFX-Ca (low dose) > EFX-Ca (medium dose) > EFX > EFX-Ca (high dose), with the corresponding cure rates of 88%, 80%, 64%, and 58%, respectively.

In general, the efficacy of EFX-Ca in the medium- and low-dose groups was higher than that of EFX in chickens infected with *E. coli*, indicating that EFX-Ca was a more appropriate therapeutic concentration as a potential veterinary drug. Under these two doses, EFX-Ca had the highest cure rate for AA and 817 infected chickens, which were 25.8% and 37.5% higher than the EFX dose group, respectively. When the high-dose EFX-Ca reacted quickly with chickens infected with *E. coli*, however, the mortality rate of chickens was relatively high. We speculated that the high-dose EFX-Ca ruptured the bacteria and released a large amount of endotoxin, further leading to the increased mortality of the chickens [40,41,42,43,44]. For these two different types of chickens, the optimal dosage of EFX-Ca was different, and too high or too low concentration was not conducive to the therapeutic effect of infected chickens. This should be given special attention and treated differently in subsequent administration of clinical medication trials.

## 3. Materials and Methods

### 3.1. Reagents and Instruments

All of the chemical reagents were of analytical grade and were commercially available. Anhydrous dextrose was purchased from Xi’wang Pharmaceutical Co., Ltd. (Zouping, China). We used all reagents without further purification. We purchased enrofloxacin (EFX) from Hangzhou Mei’bo Pharmaceutical Chemical Co., Ltd. (Hangzhou, China), Ca(NO_3_)_2_∙4H_2_O from Alfa Aesar, and 10% formalin from Beijing Solarbio Science & Technology Co., Ltd. (Beijing, China). The standard EFX as a reference was purchased from the China Veterinary Drug Supervision Institute. All the tested mice and rats were obtained from the animal experimental center of Guangxi Medical University. Five hundred 4-week-old broilers in two types (AA white feather broilers (AA) and 817 hybrid broilers (817)) were purchased from Kang’da Livestock and Poultry Co., Ltd. (Langfang, China). All experiments were performed in compliance with the policy on animal use and ethics of Guangxi Medical University, and the animals’ blood or organs were taken under anesthesia, with quiet, rapid and painless death. To be specific, both the mice and rats used in this experiment were killed by spinal dislocation and the broilers were killed by cutting the carotid artery behind the ear with a scalpel.

The EFX-Ca and EFX were dissolved in DMSO to prepare the stock solution at a concentration of 0.67 mM. We prepared tris-NaCl buffer solution (5 mM Tris, 50 mM NaCl, adjusted to pH = 7.3 by hydrochloric acid), 0.9% normal saline, bouillon culture-medium, and solid medium using double-distilled water. The solutions were all sterilized for 30 min at 121 °C before being stored in the refrigerator. A series of concentrations (50, 76, 115, 174, 264, and 400 mg/mL) of EFX-Ca and EFX were prepared by diluting the respective stock solutions with distilled water. The infrared (IR) spectra were obtained on a PerkinElmer Fourier transform-IR (FT-IR) Spectrometer. Elemental analyses (C, H, and N) were carried out on a PerkinElmer Series II CHNS/O 2400 elemental analyzer. The mass spectra were recorded on a Thermo-Finngan LCQ/AD Quadrupole Ion Trap electrospray ionization–mass spectrometer (ESI-MS). The analysis on the stability of the complex in solution was performed on an ANL404 PerkinElmer ultraviolet-visible (UV-Vis) spectrometer and the pharmacokinetic characteristics were obtained on an Agilent 1200-6410 HPLC/MS system.

### 3.2. Synthesis of [Ca(EFX)_2_(H_2_O)_4_]·H_2_O (EFX-Ca)

EFX (0.1 mmol, 0.0360 g) was dissolved in 7 mL acetone and 8 mL water and adjusted to pH 7.5 by addition of triethylamine, yielding a clear yellow solution. Ca(NO_3_)_2_∙4H_2_O (0.05 mmol, 0.0120 g) dissolved in 10 mL water was added dropwise to this solution. The mixed solution was refluxed and stirred at 65 °C for 10 h [32]. The obtained colorless and transparent resultant was filtered, allowed to stand overnight, and then allowed to slowly evaporate for several days, before the EFX-Ca precipitated into colorless and transparent crystals, from which we selected single crystals for the single X-ray crystal diffraction analysis. Then, we filtered the product again and washed it twice with anhydrous ethanol before it was dried in vacuo overnight (Yield: 75%). FT-IR (KBr, cm^−1^): 3410.06 (m, *v*O–H), 2811.69 (m, *v*Ar–H), 2966.29 (m, *v*N–H), (C=O) 1576.97 (vs, *v*C=O), 1623.96 (vs, *v*O–C=O, asym), 1391.02 (m, *v*O–C=O, sym), and 1486.59 (s, *v*C=C). Calc. for C_38_H_52_CaF_2_N_6_O_11_ (Mw = 846.93): C 53.84, H 6.14, N 9.92; Found. C 53.25, H 6.04, N 9.85%. ESI-MS: *m*/*z* 360.3 [EFX + H]^+^, 719.4 [2EFX + H]^+^, 554.2 [EFX + Ca + 2DMSO − H]^+^, 757.3 [2EFX + Ca − H]^+^ (ESI†, Figures S14 and S15).

### 3.3. X-ray Crystallography

We collected single X-ray crystal diffraction data for EFX-Ca and EFX on a Bruker APEX-II CCD diffractometer equipped with graphite monochromated Mo-Kα radiation (λ = 0.07107 nm) at room temperature. We solved the structure and refined it according to direct methods, using OLEX2 and SHELXL-97 programs [45]. We added the hydrogen atoms theoretically, riding on the concerned atoms. The crystallographic data and refinement details of the structure analyses are summarized in Table 1 and Table S1.

### 3.4. Determination on the Water Solubility of EFX-Ca

We determined the water solubility of EFX-Ca and EFX for comparison by means of UV-Vis spectroscopy, in which we monitored the maximum absorbance at 291 nm. We prepared a standard solution by dissolving the accurately weighed quantity of EFX-Ca (or EFX) into H_2_O at 35 °C to achieve a primary standard solution. We diluted this solution into a series of working solutions to measure their UV-Vis spectra. We recorded each maximum absorbance found at 291 nm to give a standard curve according to the Lambert–Beer Law, as shown in Figure 2 and Figure S2 (ESI†). Then, we comparatively measured a saturated aqueous solution of EFX-Ca (or EFX) at 35 °C and determined its corresponding concentration by fitting the standard curve.

### 3.5. Antibacterial Activity Evaluation

The clinically typical pathogens (*Staphylococcus aureus*, *Escherichia coli*, *Pseudomonas aeruginosa*, *Salmonellosis typhoidal* and *Proteus vulgaris*) were obtained from the Department of Microbiology and Immunology of Guangxi Medical University. We cultured the bacterial suspension of five pathogens in bouillon culture-medium at 37 °C, and diluted it into 0.5 McFarland Standard with bioclean normal saline (the volume ratio of each bacterial suspension and bioclean normal saline was 1:1000, and the concentration of bacterial suspension was 1~2 × 10^8^ CFU/mL). We selected EFX and EFX-Ca as the tested drugs before they were prepared to be 10^−3^ mg/mL by DMSO. We carried out the assays for MIC and MBC evaluation by doubling the dilution under sterile operation on the clean bench [46,47]. To be specific, we set the sterile test tubes numbered 1–10 for comparison according to the half-dilution method. We added the broth medium to each test tube to fix the final volume to be 2 mL. Therefore, we prepared the final concentration of EFX-Ca or EFX in the tube nos. 1~9 to be 1/4, 1/8, 1/16, 1/32, 1/64, 1/128, 1/256, 1/512, and 1/1204 μg/mL. Then, we filled each tube with 50 μL of inoculated bacteria colony, except for tube no. 9, which was the negative control. The final colony concentration in each test tube was 2 × 10^5^ CFU/mL. Tube no. 10 was set as a blank without the tested drug. The samples were cultured at 37 °C for 24 h before we obtained and observed results the next day. We repeated all tests in triplicate.

### 3.6. Acute Toxicity Study

All tests were conducted in the animal experimental center of Guangxi Medical University. Preliminary experiments were needed before starting official tests. The determination of different dosage groups (16,000, 10,556, 6964, 4595, 3031, and 2000 mg/kg) in official tests was based on the maximum dose leading to no death and the minimum dose resulting in no survival, which we obtained from the pilot study [48,49]. Male and female KM mice (5–6 weeks old, 20–23 g weight) that were allowed free access to standard diet and tap water (with no antibacterial agents) were housed in a specific pathogen-free facility with conditions of constant photo period (12 h light/12 h dark with 25–28 °C temperature and 45–65% humidity). Altogether, 210 mice were divided into 21 groups on average, and every 7 groups were set up for EFX, EFX-Ca and EFX-Na, respectively. Groups of mice (n = 10 per group, half male and half female) received different doses of the tested compounds by gavage at the same single dose of drug solution, which was uniformed to be 0.4 mg (drug solution)/10 g, but containing different concentration of tested drugs. Then, all mice were fasted for 12 h with drinking water only. We compared these mice with the control group in which the mice were not given drugs, according to the pre-experimental procedure. We closely observed the mice for 6 h after the gavage for signs of toxicity and mortality. As soon as death occurred among the maximum-dose group, we conducted an autopsy and took samples from the main organs for histopathological examination. In addition, we monitored all of the tested mice for 2 weeks to record their weight and mortality [50].

### 3.7. Histopathological Examination

After the acute toxicity test, the dead mice after oral administration and the surviving mice at the end of the observation period were promptly dissected and pathologically examined. We checked whether the organ tissues of the animals exhibited hyperemia, hemorrhage, or other abnormal changes. We dissected the important organs (heart, liver, spleen, lung, kidney, large intestine, and small intestine) of dead mice and normal mice in the high-dose group of each drug solution. Then, the main organs were fixed in 10% buffered formalin and embedded in paraffin for 1–4 h. They were histopathologically examined under the optical microscope after slicing and staining with hematoxylin-eosin (HE) staining. No fixed parameters were compared between the treatment and control groups.

### 3.8. Pharmacokinetic Assay

#### 3.8.1. Feeding and Oral Administration on the Tested SD Rats

Altogether, nine male SD rats (6–7 weeks old, 200–240 g weight) were allowed free access to standard diet and tap water (with no antibacterial agents) and were housed with conditions of constant temperature (25–28 °C) and humidity (45–65%). These rats were marked, randomly and equally divided into three groups, respectively, for EFX, EFX-Ca and control. They were further fasted for 12 h with drinking water only before the oral administration at a single dose of 10 mg/(kg·bw) (EFX) and 12 mg/(kg·bw) (EFX-Ca). We compared these rats with the control group in which the rats were not given drugs [51,52,53].

#### 3.8.2. Working Conditions of HPLC-MS

For the HPLC-MS measurement, we dissolved the standard EFX in the distilled water to prepare a 1.0 mg/mL standard working solution. Then, we dissolved EFX and the EFX-Ca in distilled water to prepare the stock solution of 1.0 and 1.2 mg/mL, respectively. The detailed experimental working conditions are listed in Table S7.

#### 3.8.3. Blood Sample Collection and Pretreatment of the Plasma Sample by HPLC-MS

We collected blood at different time points (0, 0.083, 0.333, 0.5, 0.75, 1, 3, 4, 6, 8, 10, 12, and 24 h) from the rat’s eye socket vein in rotation after oral administration. Then, we separated the plasma by centrifugation at 4 °C, 3500 r/min and stored it at −20 °C until detection.

Before the drug concentrations in plasma were detected, we first needed to obtain the optimal pretreatment of the plasma samples. We gained the specification curve according to the optimal pretreatment with the blank plasma and the standard working solution of EFX. During pretreatment, a mixture of blank plasma or medicated plasma (300 μL), hexyl hydride (2.5 mL), and dichloromethane (2.5 mL) was centrifuged for the supernatant (4.0 mL) at 1000 r/min. We dried the plasma by nitrogen blowing on a water bath at 40 °C, dissolved it with the mobile phase (300 μL), and extracted the supernatant for analysis after filtrating with 0.22 μM ultra-filtration membrane. We made a series of plasma samples with different concentrations of EFX (0.025, 0.05, 0.1, 0.5, and 1 μL/mL, which were marked on the *x*-axis) [51,52,53,54] by adding standard working solutions (30 μL) of different concentrations into the blank plasma (270 μL), and treated them in accordance with the previous method. Then, we recorded a series of peak areas (*y*-axis) by HPLC-MS, to get the relationship between the *y*- and *x*-axis. A linear equation for a standard curve of EFX, *y* = 19,373*x* − 72.512, was thus achieved (*R*^2^ = 0.9997). From the above plasma matrix standard solution, the limit of detection (LOD) of EFX was determined to be 0.1 ng/mL, based on the EFX concentration of the sample with Signal to Noise Ratio (SNR) = 3. The limit of quantitation (LOQ) of EFX was determined to be 1.0 ng/mL, based on the EFX concentration of the sample with SNR = 10. The standard curve and the relationship between drug concentration and the elimination time in plasma samples were formatted by Origin software. The main pharmacokinetic parameters, such as Ke, *T*_max_, *C*_max_, *t*_1/2_, AUC_0−t_, etc., were calculated by WinNonlin (Version 5.2.1) software, and processed by the non-compartmental model.

### 3.9. In Vivo Acute Toxicity and Anti-Inflammatory Effects on Zebrafish

#### 3.9.1. Preliminary Evaluation on the Acute Toxicity on Zebrafish

The wild type AB strain zebrafish was further used for the preliminary evaluations of EFX-Ca on the acute toxicity on zebrafish. Zebrafish were incubated and grown in the Joint Laboratory established by Shandong Rui’ying Pharmaceutical Group (Heze, China) and China Pharmaceutical University. Both the Wild-type AB (*Danio rerio*) and Tg(mpx:eGFP) zebrafish strains were maintained under the guidance of the standard procedures of The Zebrafish Book [55]. Adult zebrafish were incubated under the optimized conditions (28.5 ± 0.5 °C, pH = 7.5, light (14 h)/dark (10 h) per day) and fed brine shrimp twice a day. In order to obtain the embryos, firstly, the healthy zebrafish, female and male each half and separated by clapboard for 12 h before the experiment, were chosen the night before and were kept in the spawning tank. Then the zebrafish were allowed to naturally mate and spawn the next morning. The normally born embryos were then collected after 1 h and kept in an incubating system at 28 °C. The 6 hpf (hour post fertilization) embryos were transferred into a 6-well plate exposed to 5 mL of egg water, in which 30 embryos were in each well. Then different concentrations (0.01, 0.1, 1, 10, 20 μM) of the tested EFX-Ca, compared with EFX itself, were added into each well, along with a well set as blank control. The zebrafish’s developmental status, abnormalities (such as body length, pericardial edema, spinal curvature) and death numbers were then carefully observed under stereoscopic microscope and recorded at the time points of 6, 24, 48, 72, 96 and 120 hpf of the embryos, respectively.

#### 3.9.2. In Vivo Anti-Inflammation Assay in Zebrafish

The transgenic zebrafish line Tg(mpx:eGFP) with green fluorescence at 3 dpf (day post fertilization) were primarily used for the anti-inflammation evaluation effect of EFX-Ca. This zebrafish model was set and induced by 40 min of incubation of 10 μM of CuSO_4_. Before the CuSO_4_-induced model was build, the Tg(mpx:eGFP) zebrafish were primarily incubated with different concentrations of EFX-Ca, also in comparison with EFX for 1 h, of 0.01, 0.1 and 1 μM, respectively, based on the above results of preliminary acute toxicity evaluation. The ROS production induced by CuSO_4_ in zebrafish could then be probed and visualized by the migration and aggregation on those labeled neutrophils. The zebrafish were also photographed under the stereo microscope.

The wild-type AB zebrafish at 5 dpf (day post fertilization) were further used for another anti-inflammation model induced by H_2_O_2_ to evaluate the anti-inflammation effect of EFX-Ca. In the experiment, 15 embryos were set in each well of the 6-well plate, and the incubated concentrations of EFX-Ca, compared with EFX, were fixed at 0.01, 0.1 and 1 μM, respectively. After 48 h of incubation with different concentrations of EFX-Ca or EFX, each medicated bath solution was eluted by egg water three times, then followed by the incubation of H_2_O_2_ (1.0 mM) for 4 h. The H_2_O_2_-containing solution was eluted again and then DCFH-DA (10 µM) was added into the new egg water solution in darkness to label the potential induced ROS in zebrafish for 30 min. Then the fluorescence emission of DCF-DA oxidized by the formed ROS in zebrafish could be visualized and photographed under the stereo microscope.

### 3.10. Quasi-Clinical Trials on Broilers

We purchased 500 4-week-old broilers in two types: AA white feather broilers (AA) and 817 hybrid broilers (817) from Kang’da Livestock and Poultry Co., Ltd. Anhydrous dextrose was purchased from Xi’wang Pharmaceutical Co., Ltd. We prepared the 5% EFX and EFX-Ca mixed with anhydrous dextrose as the oral liquid for the therapeutic trial. Before the trial, all chickens that were allowed a standard diet and tap water (with no antibacterial agents) were housed for a week in a specific pathogen-free facility with conditions of illumination (24 h), desiccation, temperature (25–28 °C), and ventilation. Then, broilers were randomly and equally divided into five different groups and marked as five groups. For the AA broilers, the groups included the following: high dosage of EFX-Ca (14 g drug/1.5 kg feed), medium dosage of EFX-Ca (9 g drug/1.5 kg feed), low dosage of EFX-Ca (6 g drug/1.5 kg feed), and single dosage of EFX (11 g drug/1.5 kg feed), and the control group. It should be noted that the “g drug” in the administration unit (g drug/kg feed) refers to the drug solution containing 5% EFX-Ca or EFX. For the 817 broilers, the feed amount in each group was decreased to 0.75 kg. Note that the daily drug-mixed feeding ratio (g drug/kg feed) was indicated for the first 4 h every day, and unmedicated feed then followed. The experimental data are detailed in Table 9. In addition, *E. coli* strains for the infection of chicken was supplied as a freeze-dried powder. They were revived and cultured according to the standard procedures. The strain was inoculated into a liquid medium and cultured in a 37 °C incubator for 6–8 h. We detected the concentration of the bacterial liquid to be 2.8 × 10^8^ CFU/mL as the pathogenic concentration. Finally, all of the chickens except the healthy control group were intramuscularly injected with 0.5 mL of pathogenic bacteria, and all chickens were fasted for 4 h. After successfully simulating the bacterial infection of the chickens, the drug-mixed feeding chickens were continuously fed for 6 d. During this time, we observed their behavioral habits, mortality, and recovery rates.

## 4. Conclusions

Overall, viewed from the results above, it has been demonstrated that the combination of the appropriate metal ions and drugs could improve the efficacy of the original drug and reduce the toxicity. This calcium complex of EFX might be partly recognized, but not limited, as a modified or derived compound from EFX. However, it is indeed a new agent when introducing the bio-safe Ca(II) to EFX based on coordination chemistry, which showed superiority over EFX for lower in vivo toxicity and higher therapeutic efficacy on the tested mice and two broiler strains. Although the detailed molecular mechanism and key role of the central Ca(II) in exerting better efficacy remain to be further investigated, the present research findings demonstrated that combining the appropriate and biocompatible macrometallic element with organic veterinary drugs on the chemical level had immeasurable potential to improve therapeutic efficacy. This study indicated a promising pathway for the development of new metal-based veterinary drugs.

## Figures and Tables

**Table 1:** Crystallographic and refinement parameters for EFX-Ca.

Empirical Formula	C_38_H_53_CaF_2_N_6_O_11_
Formula weight	847.94
Temperature / *K*	296.15
Crystal system	*Monoclinic*
Space group	*C2/c*
*a*/Å, *b*/Å, *c*/Å	14.891(3), 26.250(5), 10.710(2)
*α*/°, *β*/°, *γ*/°	90.00, 102.825(3), 90.00
Volume/Å^3^	4081.9(14)
*Z*	4
*ρ*_calc_/mg mm^−3^	1.380
*μ*/mm^−1^	0.229
*F* (000)	1796
Crystal size/mm^3^	0.40 × 0.20 × 0.12
2*θ* range for data collection	3.1 to 52.74°
Index ranges	−18 ≤ *h* ≤ 18, −32 ≤ *k* ≤ 32, −13 ≤ *l* ≤ 13
Reflections collected	24,569
Independent reflections	4187 [*R*(int) = 0.0336]
Data/restraints/parameters	4187/9/279
Goodness-of-fit on *F*^2^	1.056
Final *R* indexes [*I* > 2*σ* (*I*)]	*R*_1_ = 0.0384, *wR*_2_ = 0.1116
Final *R* indexes [all data]	*R*_1_ = 0.0487, *wR*_2_ = 0.1200
Largest diff. peak/hole/*e* Å^−3^	0.360/−0.512

**Table 2:** The antibacterial activity against five bacteria of EFX and EFX-Ca shown as MIC and MBC values (μg/mL).

Type of Bacteria	EFX		EFX-Ca	
	MIC	MBC	MIC	MBC
*S. aureus*	0.25	0.25	0.25	0.25
*E. coli*	0.0625	0.0625	0.03125	0.0625
*S. typhi*	0.03125	0.0625	0.03125	0.0625
*P. aeruginosa*	0.125	0.125	0.125	0.125
*P. vulgaris*	0.125	0.125	0.125	0.125

**Table 3:** Changes in the body weight (g, $\bar{x}$ ± SD) of the tested KM mice caused by EFX-Ca at different dosages (2000, 3031, 4595, 6964, 10,556 and 16,000 mg/kg).

Group	Dose (mg/kg)	$\mathrm{Body}\;\mathrm{Weight}\;(g,\;\bar{x}$ ± SD)				
		Day-0	Day-1	Day-4	Day-7	Day-14
Control	--	17.21 ± 1.25	21.57 ± 1.37	27.03 ± 1.53	30.47 ± 2.61	35.00 ± 5.19
1	16,000	17.84 ± 1.23	17.43 ± 1.95 *	23.55 ± 2.66	29.15 ± 2.66	37.25 ± 4.57
2	10,556	17.67 ± 0.72	17.00 ± 0.30 *	21.53 ± 2.10 *	26.47 ± 3.50 *	34.00 ± 7.50
3	6964	17.68 ± 0.88	17.21 ± 1.22 *	16.89 ± 5.13 *	23.11 ± 4.21 *	29.30 ± 5.26 *
4	4595	17.31 ± 1.05	17.09 ± 1.56 *	20.10 ± 5.55 *	26.43 ± 5.34 *	33.60 ± 7.07
5	3031	18.32 ± 0.94	19.61 ± 1.23 *	25.70 ± 1.24	28.44 ± 2.14	33.91 ± 5.25
6	2000	17.51 ± 1.14	19.77 ± 1.22 *	25.91 ± 1.83	29.78 ± 3.46	33.33 ± 5.64

Note: * *p* < 0.05 (compared with control); The total number of the tested KM mice was 70.

**Table 4:** The acute toxicity test on KM mice administrated different dosages (2000, 3031, 4595, 6964, 10,556 and 16,000 mg/kg) of EFX-Ca, compared with EFX.

**EFX-Ca**						
Dose (mg/kg)	2000	3031	4595	6964	10,556	16,000
Death number	0	3	4	3	7	10
**EFX**						
Dose (mg/kg)	2000	3031	4595	6964	10,556	16,000
Death number	2	5	5	4	9	10

**Table 5:** Main pharmacokinetic parameters of the plasma sample from the tested SD rats under the oral administration of EFX and EFX-Ca, respectively.

Parameter	Unit	EFX (10 mg/kg)	EFX-Ca (12 mg/kg)
*C*_max_	ng/mL	830	1920
*T_max_*	H	1	0.75
*t*_1/2β_	H	2.43	4.41
AUC_0−t_	ng·h/mL	2702.50	5875.83
Ke	---	0.28	0.16
Vd	mL/kg	1053.16	267.44
Cl	mL/kg·h	37.00	20.42

**Table 6:** The statistical survival (death) of the tested zebrafish under the medicated bath of different concentrations (0.01, 0.1, 1, 10, 20 μM) of EFX-Ca or EFX.

	**EFX-Ca**					
	**Control**	**0.01**	**0.1**	**1**	**10**	**20**
24 hpf	28(2)	24(6)	26(4)	28(2)	26(4)	30(0)
48 hpf	28(2)	24(6)	26(4)	28(2)	26(4)	30(0)
72 hpf	28(2)	24(6)	26(4)	28(2)	26(4)	30(0)
96 hpf	28(2)	22(8)	26(4)	28(2)	26(4)	30(0)
120 hpf	28(2)	22(8)	26(4)	28(2)	26(4)	30(0)
	**EFX**					
	**Control**	**0.01**	**0.1**	**1**	**10**	**20**
24 hpf	28(2)	24(6)	26(4)	28(2)	28(2)	26(4)
48 hpf	28(2)	24(6)	26(4)	28(2)	28(2)	26(4)
72 hpf	28(2)	24(6)	26(4)	28(2)	28(2)	26(4)
96 hpf	28(2)	24(6)	26(4)	28(2)	28(2)	26(4)
120 hpf	28(2)	24(6)	26(4)	28(2)	28(2)	26(4)

**Table 7:** The survival numbers, cure rates, and relative weight gain rates on two typical broilers after drug-mixed feeding of EFX and different dosages of EFX-Ca, respectively.

Type of Broilers	Group	Number	Survival Number	Cure Rate (%)	Average Weight Gain	Relative Weight Gain Rate (%)
AA	Control	50	50	100	730	100
	EFX	50	31	62	380	52
	EFX-Ca (High)	50	30	60	250	34
	EFX-Ca (Medium)	50	39	78	140	19
	EFX-Ca (Low)	50	27	54	190	26
817	Control	50	50	100	600	100
	EFX	50	32	64	100	17
	EFX-Ca (High)	50	29	58	70	12
	EFX-Ca (Medium)	50	40	80	80	13
	EFX-Ca (Low)	50	44	88	280	47

Note: Relative weight gain rate = (the average weight gain per dose/weight gain in the healthy control group) × 100%.

**Table 8:** The influence on the body weight, feed consumption and feed conversion ratios on two typical broilers after drug-mixed feeding of EFX and different dosage of EFX-Ca, respectively.

Type of Broilers	Group	Average Initial Weight (g)	Average Final Weight (g)	Average Increment of Weight (g)	Total Feed Consumption (g)	Average Feed Consumption (g)	Feed Conversion Ratio (FCR)
AA	Control	1500 ± 68	2230 ± 104	730	67,000	1340	1.84:1
	EFX	1630 ± 120	1880 ± 113	250	13,200	264	1.06:1
	EFX-Ca (High)	1520 ± 78	1750 ± 125	230	15,000	300	1.30:1
	EFX-Ca (Medium)	1360 ± 57	1640 ± 73	280	16,000	320	1.14:1
	EFX-Ca (Low)	1500 ± 94	1690 ± 95	190	11,000	220	1.16:1
817	Control	930 ± 71	1530 ± 74	600	51,000	1020	1.7:1
	EFX	890 ± 56	1030 ± 74	140	7300	146	1.04:1
	EFX-Ca (High)	920 ± 58	1000 ± 51	80	4500	90	1.13:1
	EFX-Ca (Medium)	870 ± 47	1010 ± 61	140	7500	150	1.07:1
	EFX-Ca (Low)	950 ± 56	1210 ± 67	260	14,000	280	1.08:1

**Table 9:** Conditions for the drug-mixed feeding on two types of tested broilers.

Type of Broilers	Conditions	Compound in Each Group	Number	Dosage (Drug in Solution + Feed)/4 h
AA	Uninfected	Control	50	1.5 kg
	infected	EFX	50	11 g + 1.5 kg
	infected	EFX-Ca (High)	50	14 g + 1.5 kg
	infected	EFX-Ca (Medium)	50	9 g + 1.5 kg
	infected	EFX-Ca (Low)	50	6 g + 1.5 kg
817	Uninfected	Control	50	0.75 kg
	infected	EFX	50	11 g + 0.75 kg
	infected	EFX-Ca (High)	50	14 g + 0.75 kg
	infected	EFX-Ca (Medium)	50	9 g + 0.75 kg
	infected	EFX-Ca (Low)	50	6 g + 0.75 kg

Note: For each group, each broiler was orally gavaged the medicated feed for the first 4 h daily and then followed by unmedicated feed. The feed was mixed with 5% drug contained in an aqueous solution, in which 11 mg EFX or 14, 9, 6 mg EFX-Ca, respectively, was added for each broiler. The unit in the last column (g drug in solution/kg feed) refers to the medicated aqueous solution containing 5% EFX-Ca or EFX.

## Data Availability

The data presented in this study are available in this article and related Supplementary Material. CCDC No. 2013726 for the calcium(II) complex of enrofloxacin, EFX-Ca, contains the supplementary crystallographic data for this paper. The data can be obtained free of charge via http://www.ccdc.cam.ac.uk, accessed on 15 January 2022, or from the Cambridge Crystallographic Data Centre, 12 Union Road, Cambridge CB21EZ, UK (Fax: +44-1223-336-033; E-mail: deposit@ccdc.cam.ac.uk).

## Supplementary Materials

The following supporting information can be downloaded at: https://www.mdpi.com/article/10.3390/pharmaceutics14020249/s1, Figure S1: The packing diagram of the crystal structure of EFX-Ca viewed along the b-axis showing the C–H···O hydrogen bonding (indicated by the dashed lines) as well as the π–π stacking interactions between the neighboring EFX ligands; Figure S2: The water solubility of EFX at room temperature was determined by the Lambert-Beer’s Law based on the standard line of the water solubility of EFX derived from a series of prepared d concentrations. The concentration of the working solution of EFX indicated by the UV-Vis spectrum was based on a 30× dilution on the saturated aqueous solution of EFX; Figure S3: The time-dependent effects of the oral administration of EFX on the body weight of the tested KM mice; Figure S4: The time-dependent effects of the oral administration of EFX-Na on the body weight of the tested KM mice; Figure S5: The standard curve recorded by HPLC for the relationship between the concentrations and the peak area of EFX in the tested SD rats; Figure S6: The survival rate of the tested zebrafish under the medicated bath of different concentrations (0.01, 0.1, 1, 10, 20 µM) of EFX-Ca or EFX; Figure S7: The preliminary acute toxicity evaluation of EFX-Ca, compared with EFX, at same concentrations (0.01, 0.1, 1 µM) on zebrafish and observed directly by the stereo microscope; Figure S8: The other two examined zebrafish with similar results of the inhibition effect of EFX-Ca, on the observed neutrophil cluster aggregation under the CuSO4-induced mode of the transgenic zebrafish line Tg (mpx: eGFP); The partial magnified images of the latter part of the zebrafish were shown on the right side; Figure S9: The other two examined zebrafish with similar results of the inhibition effect of EFX, on the observed neutrophil cluster aggregation under the CuSO_4_-induced mode of the transgenic zebrafish line Tg (mpx: eGFP); The partial magnified images of the latter part of the zebrafish were shown on the right side; Figure S10: The other two examined zebrafish with similar results of the anti-inflammatory effect of EFX-Ca on the H_2_O_2_-induced ROS production in zebrafish model, indicated by the green fluorescence probed and visualized by the added DCFH-DA; Figure S11: The other two examined zebrafish with similar results of the anti-inflammatory effect of EFX on the H_2_O_2_-induced ROS production in zebrafish model, indicated by the green fluorescence probed and visualized by the added DCFH-DA; Figure S12: General clinical observations on the infected chicken showing the characteristic pathological features (C, D, E, F) during the oral administration of EFX-Ca, compared with the uninfected chicken with normal symptoms (A, B); Figure S13: The partial pathologic anatomy of the infected chickens that died after the oral administration of EFX-Ca; Figure S14: The IR spectrum of EFX-Ca; Figure S15: The ESI-MS spectrum of EFX-Ca; Table S1: The selected bond lengths (Å) and bond angles (°) for EFX-Ca; Table S2: Changes in body weight of KM mice caused by EFX (g, ±s); Table S3: Changes in body weight of KM mice caused by EFX-Na (g, ±s); Table S4: The relationship between the concentrations and the corresponding peak area of EFX in the tested SD rats recorded by HPLC; Table S5: Drug concentration in plasma at different time points after the administration of EFX and EFX-Ca in the tested SD rats; Table S6: The working conditions for HPLC-MS.

## Abbreviations

AUC: Area Under Curve; CCD: charge-coupled device; CFU: colony forming unit; DMSO: dimethyl sulfoxide; *E. coli*: *Escherichia coli*; EFX: Enrofloxacin; EFX-Ca: The calcium(II) complex of enrofloxacin; EFX-Na: The sodium salt of enrofloxacin; ESI-MS: Electrospray ionization–mass spectrometer; FQN: Fluoroquinolone; FT-IR: Fourier Transform Infrared Spectroscopy; HE: Hematoxylin-eosin; HPLC-MS: High Performance Liquid Chromatography-Mass Spectrometry; IOD: Integrated Optical Density; KM mice: Kunming mice; LD_50_: median lethal dose on fifty percent; LOD: limit of detection; LOQ: limit of quantitation; MBC: minimum bactericidal concentration; MIC: minimum inhibitory concentration; *P. aeruginosa*: *Pseudomonas aeruginosa*; *P. vulgaris*: *Proteus vulgaris*; *S. aureus*: *Staphylococcus aureus*; *S. Typhi*: *Salmonellosis typhoidal*; SNR: Signal to Noise Ratio; ROS: reactive oxygen species; UV-Vis: Ultraviolet-visible.
