# Supplementary material for: A New Calcium(II)-Based Substitute for Enrofloxacin with Improved Medicinal Potential
Source: Pharmaceutics. 2022 Jan 21;14(2):249. doi: 10.3390/pharmaceutics14020249 (PMC8878047; doi:10.3390/pharmaceutics14020249)
Supplement: Supplementary file 1 [file pharmaceutics-14-00249-s001.zip › pharmaceutics-1485017-supplementary.pdf]

Supplementary materials: A New Calcium(II)-Based Substitute for Enrofloxacin with Improved Medicinal Potential

Hou-Tian Yan, Rui-Xue Liu, Qi-Zhen Yang, Yan-Cheng Liu, Hong-Chang Li, Rui-Feng Guo, Lin-Hua Wu, Li-Min Liu and Hong Liang

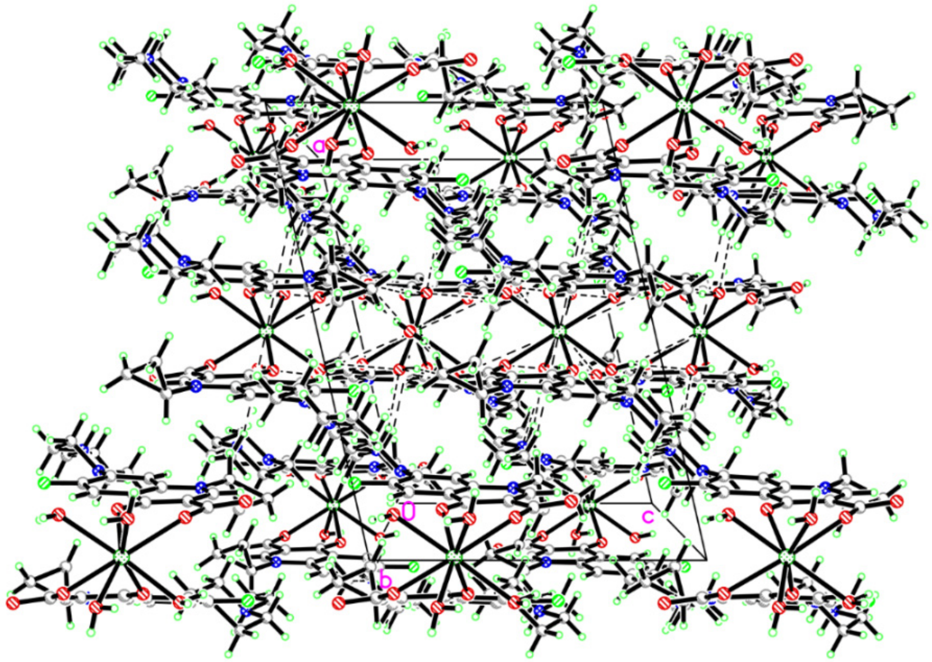

Figure S1. The packing diagram of the crystal structure of EFX-Ca viewed along the *b*-axis showing the C–H···O hydrogen bonding (indicated by the dashed lines) as well as the π–π stacking interactions between the neighboring EFX ligands.

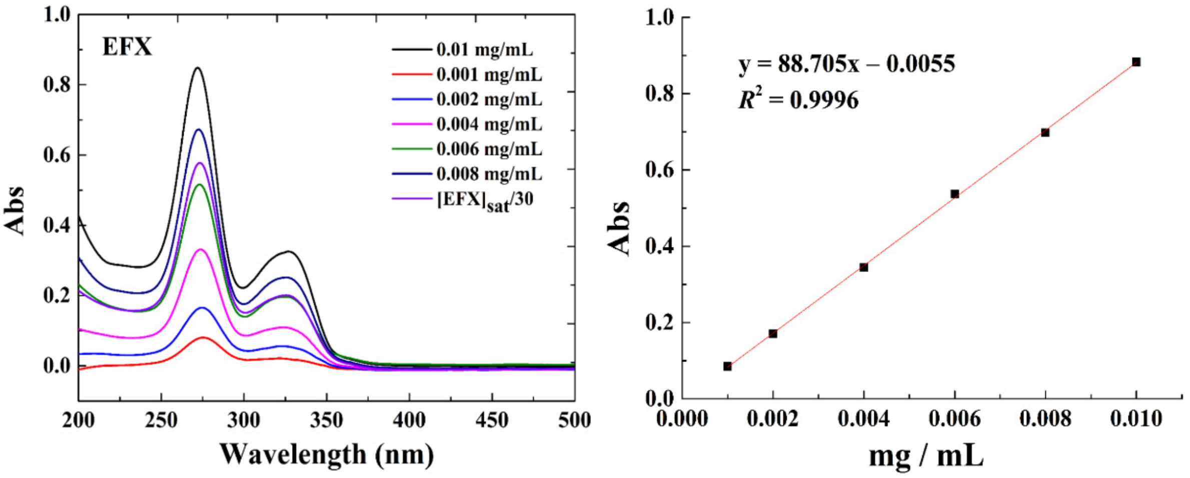

Figure S2. The water solubility of EFX at room temperature was determined by the Lambert-Beer's Law based on the standard line of the water solubility of EFX derived from a series of prepared d concentrations. The concentration of the working solution of EFX indicated by the UV-Vis spectrum was based on a 30× dilution on the saturated aqueous solution of EFX.

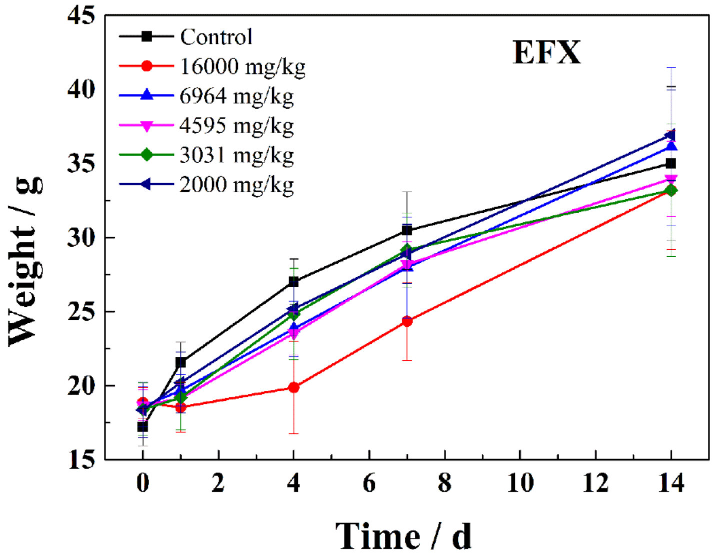

Figure S3. The time-dependent effects on the body weight of the tested KM mice after the oral administration of EFX at different dosages (2000, 3031, 4595, 6964 and 16,000 mg/kg).

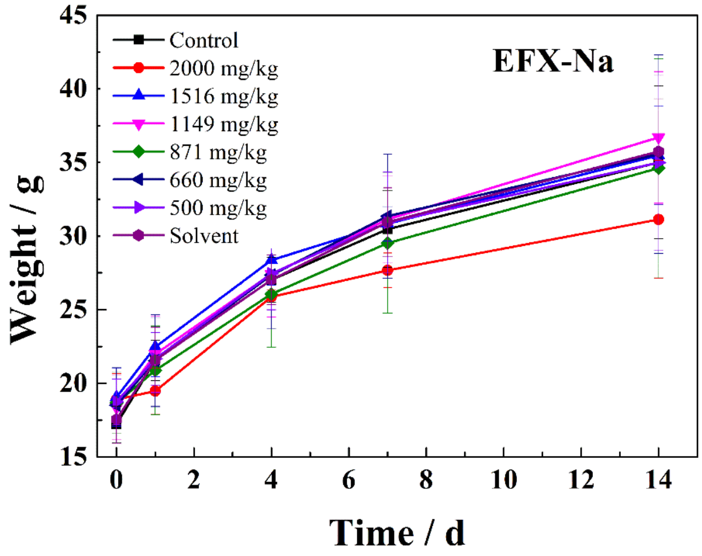

Figure S4. The time-dependent effects on the body weight of the tested KM mice after the oral administration of EFX-Na at different dosages (500, 660, 871, 1149, 1516 and 2000 mg/kg).

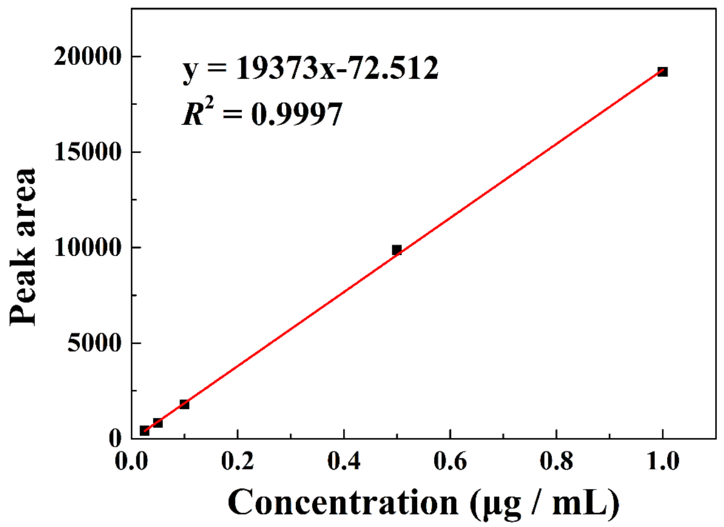

Figure S5. The standard curve recorded by HPLC for the relationship between the concentrations and the peak area of EFX in the tested SD rats.

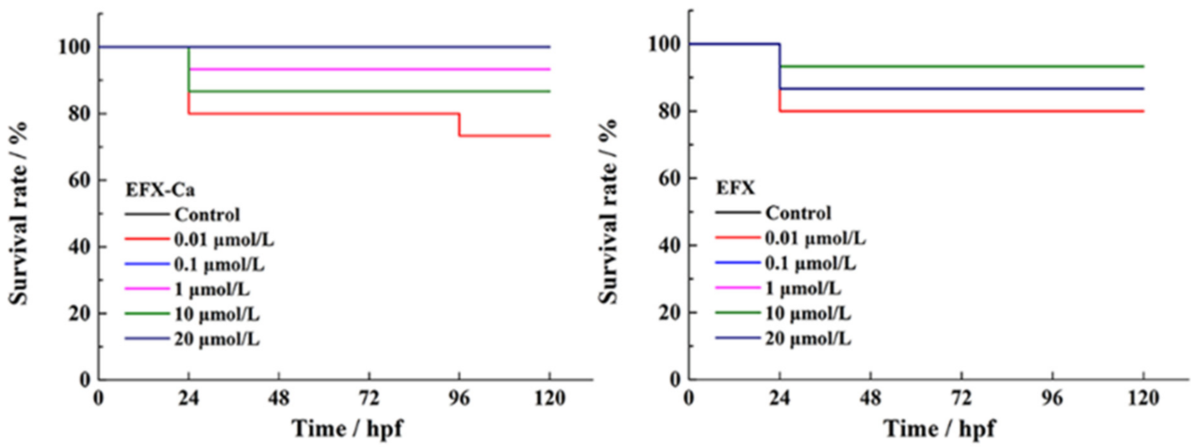

Figure S6. The survival rate of the tested zebrafish under the medicated bath of different concentrations (0.01, 0.1, 1, 10, 20 μM) of EFX-Ca or EFX.

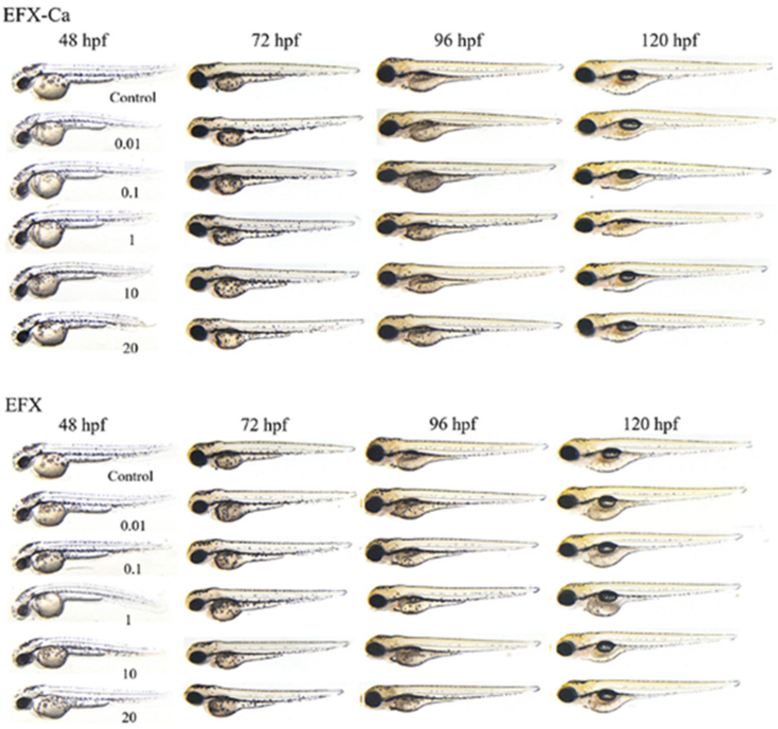

Figure S7. The preliminary acute toxicity evaluation of EFX-Ca, compared with EFX, at same concentrations (0.01, 0.1, 1 μM) on zebrafish and observed directly by the stereo microscope.

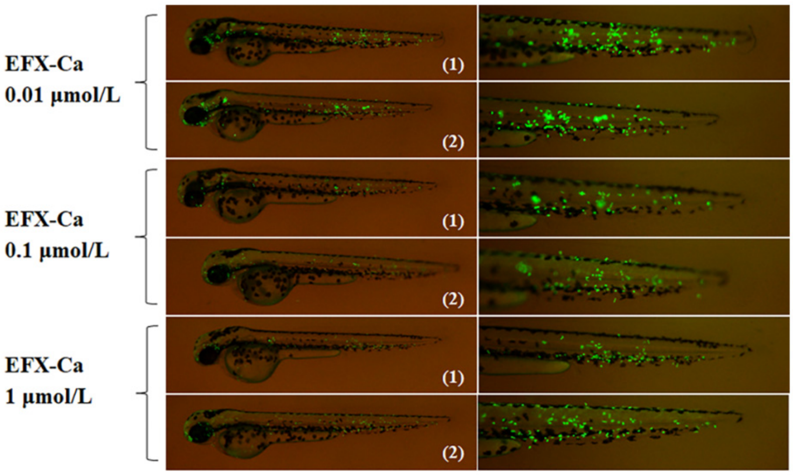

Figure S8. The other two examined zebrafish with similar results of the inhibition effect of EFX-Ca, on the observed neutrophil cluster aggregation under the $CuSO_4$-induced mode of the transgenic zebrafish line Tg (mpx: eGFP); The partial magnified images of the latter part of the zebrafish were shown on the right side.

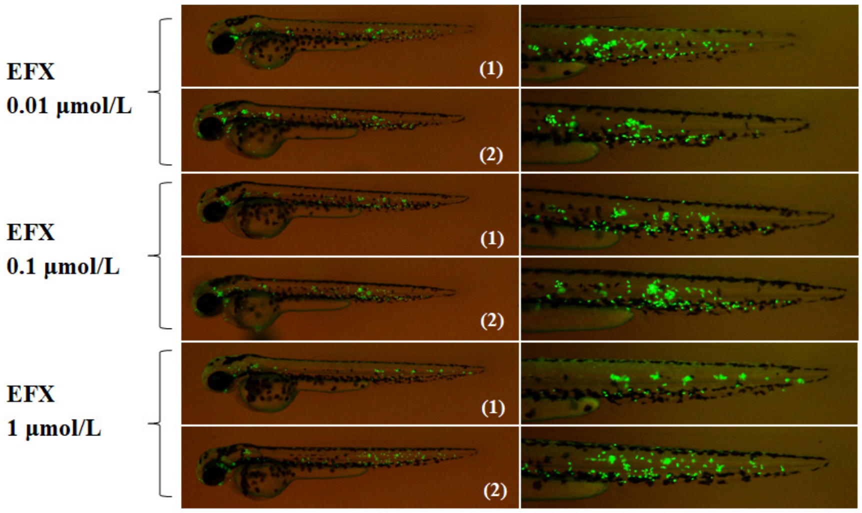

Figure S9. The other two examined zebrafish with similar results of the inhibition effect of EFX, on the observed neutrophil cluster aggregation under the $CuSO_4$-induced mode of the transgenic zebrafish line Tg (mpx: eGFP); The partial magnified images of the latter part of the zebrafish were shown on the right side.

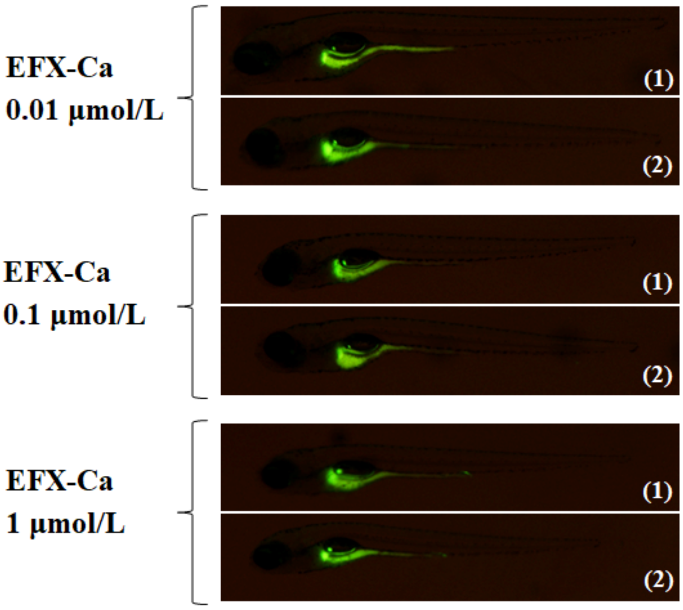

Figure S10. The other two examined zebrafish with similar results of the anti-inflammatory effect of EFX-Ca on the H_2O_2-induced ROS production in zebrafish model, indicated by the green fluorescence probed and visualized by the added DCFH-DA.

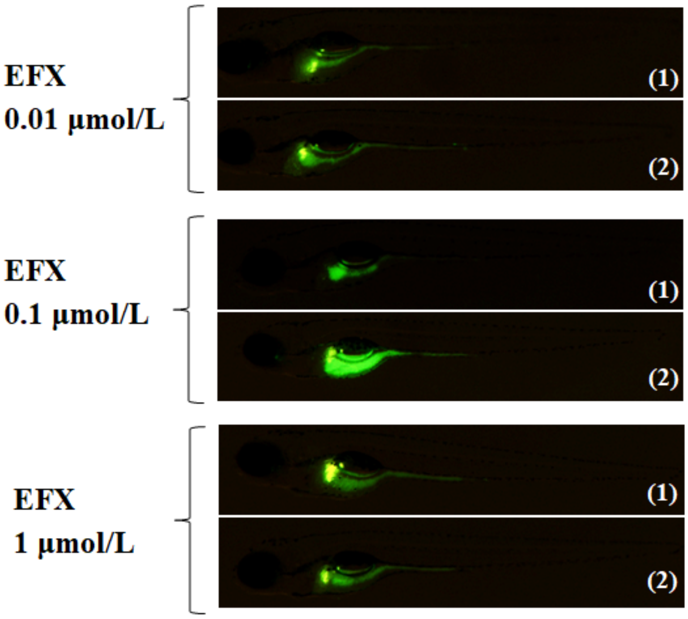

Figure S11. The other two examined zebrafish with similar results of the anti-inflammatory effect of EFX on the H_2O_2-induced ROS production in zebrafish model, indicated by the green fluorescence probed and visualized by the added DCFH-DA.

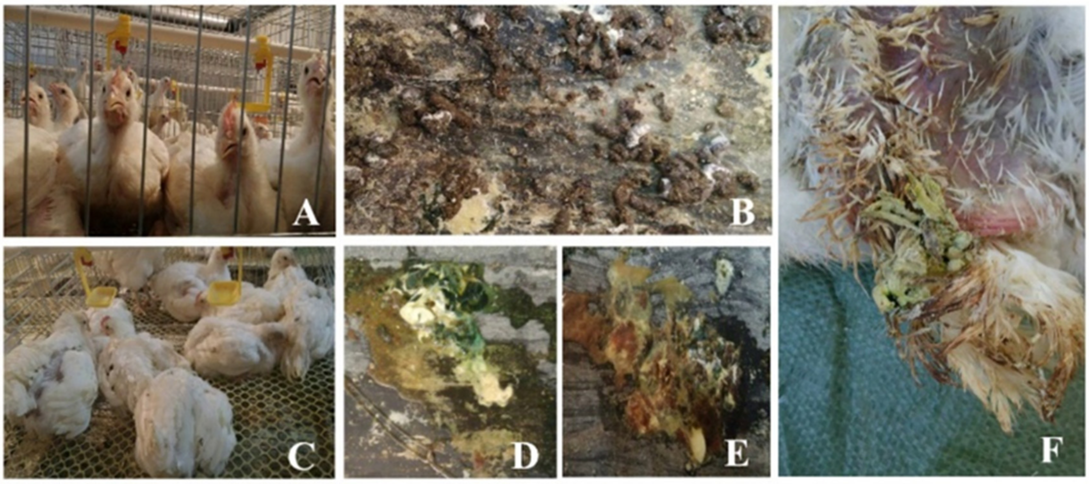

Figure S12. General clinical observations on the infected chicken showing the characteristic pathological features (**C**, **D**, **E**, **F**) during the oral administration of EFX-Ca, compared with the uninfected chicken with normal symptoms (**A**, **B**).

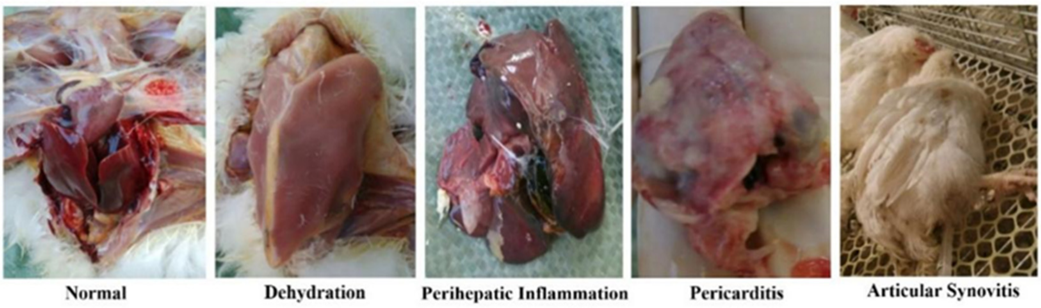

Figure S13. The partial pathologic anatomy of the infected chickens that died after the oral administration of EFX-Ca.

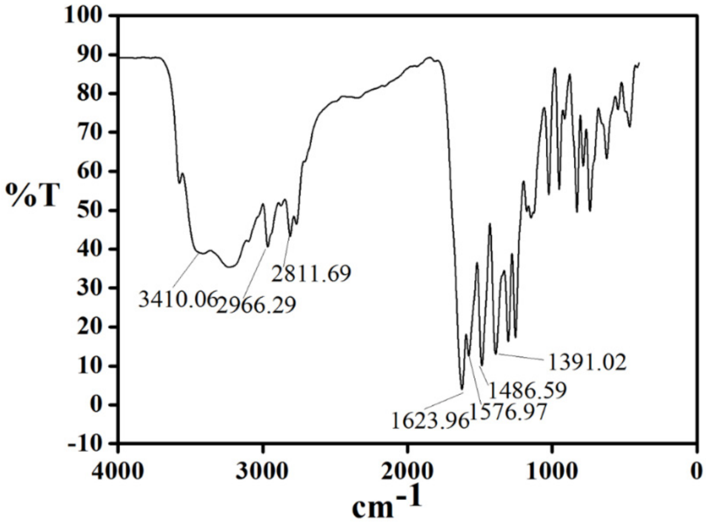

Figure S14. The IR spectrum of EFX-Ca

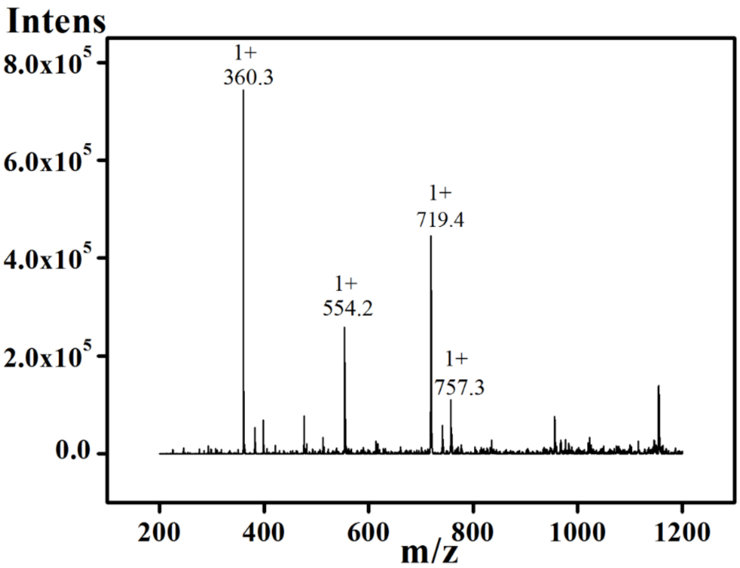

Figure S15. The ESI-MS spectrum of EFX-Ca.

Table S1. The selected bond lengths (Å) and bond angles (°) for EFX-Ca.

**Selected bond (Å)**			
Ca-O1	2.4638(12) Å	Ca-O4	2.4445(13) Å
Ca-O2	2.4080(13) Å	Ca-O5	2.5490(14) Å
**Selected angles (°)**			
O1-Ca-O1[a]	72.18(6) °	O2[a]-Ca-O4	72.13(5) °
O1[a]-Ca-O5	112.44(5) °	O2[a]-Ca-O5	75.93(4) °
O1-Ca-O5	159.09(4) °	O2-Ca-O5	129.71(4) °
O2[a]-Ca-O1	87.44(5) °	O4-Ca-O1[a]	128.72(4) °
O2-Ca-O1	69.63(4) °	O4-Ca-O1	73.27(4) °
O2-Ca-O2[a]	151.87(6) °	O4[a]-Ca-O4	155.89(7) °
O4-Ca-O5[a]	70.76(5) °	O4-Ca-O5	89.39(5) °
O5-Ca-O5[a]	70.98(7) °	O2-Ca-O4	114.10(5) °

Table S2 Changes in the body weight (g, $\bar{x} \pm$ SD) of the tested KM mice caused by EFX at different dosages (2000, 3031, 4595, 6964 and 16,000 mg/kg).

**Group**	**Dose (mg/kg)**	**Body Weight (g, $\bar{x} \pm$ SD)**				
		**d0**	**d1**	**d4**	**d7**	**d14**
Control	--	17.21±1.25	21.57±1.37	27.03±1.53	30.47±2.61	35.00±5.19
1	16,000	18.86±1.03*	18.55±1.70	19.88±3.12	24.35±2.63	33.20±4.00
2	10,556	ND	ND	ND	ND	ND
3	6964	18.59±1.34*	19.68±1.06	23.85±1.88	27.98±3.40	36.13±5.35
4	4595	18.67±1.05*	19.14±0.96	23.54±1.44	28.20±1.52	33.96±2.53
5	3031	18.40±1.78	19.20±2.18	24.83±3.08	29.17±2.51	33.20±4.46
6	2000	18.35±1.87	20.21±2.06	25.19±1.81	28.89±1.99	36.93±3.07

Note: * $P < 0.05$ (compared with control); The total number of the tested KM mice was 70.

Table S3. Changes in the body weight (g, $\bar{x} \pm SD$) of the tested KM mice caused by EFX-Na at different dosages (500, 660, 871, 1149, 1516 and 2000 mg/kg).

Group	Dose (mg/kg)	Body Weight (g, $\bar{x} \pm SD$)				
		d0	d1	d4	d7	d14
Control	--	17.21±1.25	21.57±1.37	27.03±1.53	30.47±2.61	35.00±5.19
1	2000	18.92±1.74	19.47±1.58	25.87±0.50	27.67±1.18	31.13±4.00
2	1516	19.06±1.98*	22.46±2.02	28.36±1.69	30.84±1.17	35.48±3.33
3	1149	18.35±2.18*	21.98±2.59	27.42±2.90	31.14±2.93	36.70±4.47
4	871	18.66±2.08*	20.88±3.01	26.06±3.60	29.52±4.76	34.61±7.45
5	660	18.50±2.57*	21.54±3.12	27.35±3.66	31.36±4.22	35.56±6.74
6	500	18.74±1.55*	21.66±1.78	27.45±2.45	30.92±3.43	34.99±5.93
Solvent(Na_2CO_3)		17.55±1.61*	21.57±2.23	27.03±1.69	30.94±2.34	35.75±3.59

Note: * $P < 0.05$ (compared with control); The total number of the KM mice was 80.

Table S4. The relationship between the concentrations and the corresponding peak area of EFX in the tested SD rats recorded by HPLC.

Concentration (μg/mL)	Peak area
0.025	418
0.05	815
0.1	1802
0.5	9869
1	19183

Table S5. Drug concentration in plasma at different time points after the administration of EFX and EFX-Ca in the tested SD rats.

Time (h)	Concentration of Drugs (mg/L)	
	EFX	EFX-Ca
0	0.06	0.08
0.083	0.49	0.86
0.333	0.55	0.84
0.5	0.51	1.69

0.75	0.7	1.92
1	0.83	1.39
3	0.38	0.77
4	0.3	0.7
6	0.27	0.58
8	0.25	0.48
10	0.11	0.10
12	0.08	0.14
24	0.07	0.09

Table S6 The quantitative results for the oxidative stress in zebrafish, represented by the histogram on the right side and statistically analyzed based on examining the integrated optical density (IOD) of the green fluorescence in the tested zebrafish, three zebrafish for each group.

**Group**	**Concentration (μM)**	**IOD ($\bar{x}$±SD)**	
		**Average IOD**	**SD**
Control	--	590	49
H_2O_2-induced	--	17,250	714
	0.01	1060	162
EFX-Ca	0.1	721	77
	1.0	863	114
	0.01	970	194
EFX	0.1	772	154
	1.0	848	83

Table S7. The working conditions for HPLC-MS.

**HPLC**		**MS**	
Column	Agilent ZORBAX Eclipse XDB-C18 (4.6 min × 150 mm, 5 μm)	Scanning mode	positive ionized
Mobile phase	acetonitrile: 0.1% formic acid (*v*/*v*, 45:55)	m/z	60.2 >316.2 (quantitative ion), 360.2 >245.1 (qualitative ion)
Column temperature	30 °C	Extraction / Ionization voltage	135 V/4000 V
Flow rate	1.0 mL/min	Collision energy	15 V
UV wavelength	278 nm	Carrier gas temperature	300°C
Injection volume	20.0 μL	Speed	6 L/min
